# Hybrid Edge–Cloud Asymmetric Analytics for Portable Multimodal BCI Biosensors

**DOI:** 10.3390/bios16070394

**Published:** 2026-07-21

**Authors:** Sayantan Ghosh, Padmanabhan Sindhujaa, Pradakshana Senthil Kumar, Anand Mohan, Pachaiyappan Mahalakshmi, Balázs Gulyás, Domokos Máthé, Parasuraman Padmanabhan

**Affiliations:** 1 Department of Biophysics and Radiation Biology, Semmelweis University, 1085 Budapest, Hungary; sayantan7@gmail.com (S.G.);; 2 Department of Integrative Biology, Vellore Institute of Technology, Vellore 632014, India; 3 Department of General Medicine, PSG Institute of Medical Sciences & Research, Coimbatore 641004, India; 4 Department of Artificial Intelligence and Data Science, Panimalar Engineering College, Chennai 600123, India; 5 Department of Instrumentation, Vellore Institute of Technology, Vellore 632014, India; 6 Cognitive Neuroimaging Centre, Experimental Medicine, Nanyang Technological University, Singapore 636921, Singapore; 7 Department of Clinical Neuroscience, Karolinska Institute, 17176 Stockholm, Sweden; 8 Lee Kong Chian School of Medicine, Nanyang Technological University, Singapore 636921, Singapore; 9 Division of Neuroradiology, Department of Medical Imaging, Faculty of Medicine, Semmelweis University, 1083 Budapest, Hungary

**Keywords:** brain–computer interface, portable biosensors, multimodal biosignals, EEG, EMG, edge–cloud analytics, embedded TinyML inference, physiological state monitoring, multimodal fusion, digital health

## Abstract

Portable biosensor hardware can now sustain continuous multimodal physiological acquisition at the edge, yet the analytical layer that converts raw signals into deployment-consistent inference remains the main bottleneck for practical embedded systems. This study addresses that bottleneck by presenting the machine-learning layer of the Real-time Cognitive Grid, the analytical companion to the previously reported hardware architecture, which equips a fixed-wiring biosensor assembly with real-time physiological-state classification through an asymmetric edge–cloud workflow. The proposed framework assigns analytical responsibility across tiers: a locked 17-feature schema comprising 5 EMG features, 6 EEG spectral features, 2 cross-modal features, 2 HRV features, 1 EOG feature, and 1 EEG quality indicator governs window-bounded inference on the Arduino Nano RP2040 Connect with an LDA edge artefact requiring approximately 716 B RAM, whereas the cloud tier supports public-dataset pretraining, hardware-aligned refinement, multimodal fusion, deployment comparison, and feature-importance analysis under the same schema contract. To evaluate analytical consistency across physiological diversity, five public repositories covering stress physiology (WESAD), affective EEG (DEAP), inertial activity recognition (PAMAP2), sEMG gesture decoding (EMG Gestures), and motor-imagery EEG (EEGMMIDB) were evaluated under subject-disjoint GroupKFold (k = 5) protocols. To test whether the same contract survives translation to the physical rig, the hardware branch was evaluated under session-disjoint GroupKFold across five bench-acquired sessions. Unimodal performance was strongest in sEMG- and IMU-dominant tasks, whereas multimodal fusion improved macro-F1 by up to 0.141 over the strongest unimodal baseline in WESAD and by 0.109 in PAMAP2. In the hardware branch, the deployed edge LDA artefact reached 0.9435 macro-F1 with 0.9470 accuracy, while the retained cloud Random Forest reached 0.8792 macro-F1 with 0.8799 accuracy; feature-importance analysis further showed that the final 17-feature branch was dominated by EMG descriptors, with EEG spectral terms contributing secondary support and hardware-exclusive variables remaining weak under the present bench regime. These results show that a compact multimodal sensing assembly can be elevated beyond passive signal capture into an intelligent portable biosensor that performs context-aware interpretation with minimal user intervention, supported by a reproducible analytical workflow that remains coherent across heterogeneous benchmark repositories, hardware-specific refinement, and microcontroller-class deployment, thereby establishing cross-session bench feasibility as a structured basis for future multi-subject wearable validation.

## 1. Introduction

The rapid maturation of flexible and skin-conformable biosensor platforms, including electrochemical patches, textile-integrated electrodes, and soft electronic skins, has expanded the feasibility of continuous physiological sensing beyond conventional clinical environments and into portable, body-proximate monitoring settings [1,2]. Compact platforms can now acquire electrophysiological, autonomic, and contextual signals with increasing continuity and accessibility [3,4]. The present work addresses that analytical gap directly.

**Figure 1:**
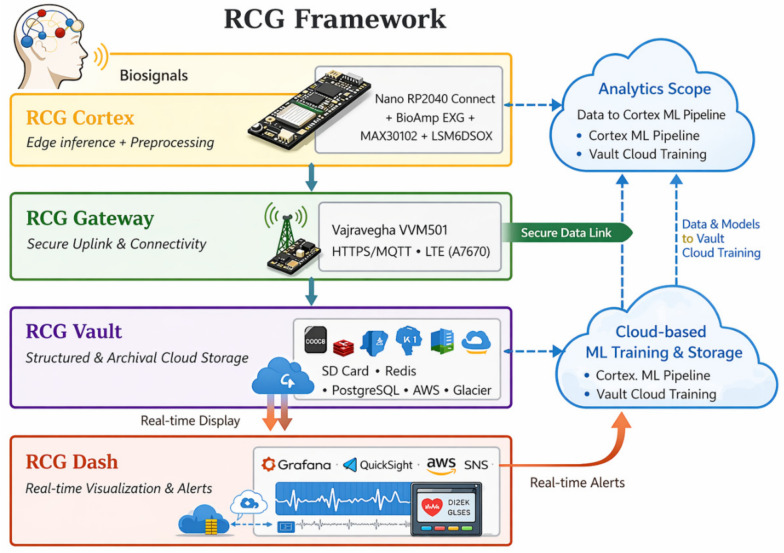
RCG framework layer architecture. Cortex: edge inference on Arduino Nano RP2040 Connect; Gateway: LTE uplink via Vajravegha VVM501; Vault: tiered storage (SD card → Redis → PostgreSQL → S3 → Glacier); Dash: visualisation and alerting. The analytics scope of the present study spans the Cortex ML pipeline and cloud-side model training, management, and analytical persistence within the Vault layer.

The previously reported Real-time Cognitive Grid (RCG) hardware architecture [5] establishes the physical platform for biosignal acquisition via four synchronous channels (sEMG, EEG, ECG, EOG), LTE gateway communication through the VVM501 ESP32 4G LTE module (Vajravegha Mobility Pvt. Ltd., Thane, India), and structured cloud storage, collectively providing the underlying hardware architecture, as depicted in Figure 1. The present analytical framework operates on this established acquisition, communication, and storage architecture, as explained in this study.

As established in the companion architecture study, the framework comprises four modular layers, namely Cortex, Gateway, Vault, and Dash. Within this broader stack, the present study is confined to the machine-learning pathway spanning edge-side inference preparation in the Cortex layer and cloud-side training, model management, and analytical persistence in the Vault layer. Figure 1, therefore, serves not as a hardware schematic, but as a boundary marker for the analytical contribution of the present paper, distinguishing the machine-learning workflow addressed here from the wider communication, storage, and interface architecture described elsewhere.

Subsequently, this study supplies the missing analytical layer by determining how multimodal physiological signals should be represented, adapted, fused, and deployed so that the embedded node functions as a state-aware inference system rather than a passive acquisition endpoint.

### 1.1. Background: Data-Driven Evolution of Wearable Biosensor Systems

Recent developments in skin-inspired bioelectronics further demonstrate that wearable sensing performance depends on system-level coordination among compliant sensing elements, interconnects, wireless communication, power delivery, and encapsulation. Consequently, acquisition hardware and downstream analytics should be treated as mutually constrained layers of a wearable system rather than as independent technological components [6,7]. In this broader trajectory, wearable and body-proximal platforms are increasingly valued not only for signal access but also for their potential to support continuous, low-obtrusion physiological analytics beyond conventional laboratory workflows. As these systems incorporate increasing numbers of sensing modalities, their analytical value depends not only on the quality of signal acquisition but also on the structure of the downstream learning pipeline that converts raw measurements into stable physiological inference [8]. This dependence is particularly pronounced in BCI-oriented contexts, where electrophysiological signals are low-amplitude, noisy, non-stationary, and strongly influenced by motion, context, and inter-subject variability, thereby complicating reliable deployment beyond tightly controlled laboratory settings [9].

Recent medical–engineering analyses of BCI acquisition systems show that electrode configuration, channel topology, signal conditioning, and recording context directly delimit the representations available to downstream classifiers. Classification performance must therefore be interpreted as a coupled consequence of acquisition architecture and analytical design, rather than as an isolated property of the selected learning algorithm. That analytical model became progressively less adequate as biosensor systems moved toward continuous monitoring, ambulatory use, and real-world inference. Under these conditions, physiological signals exhibit substantial non-stationarity, long-term drift, artefact contamination, and inter-subject variation, all of which weaken the assumptions underlying static processing pipelines [10,11].

Multimodal sensing further increases this analytical burden because neural, muscular, autonomic, and contextual streams differ in temporal scale, spectral structure, signal quality, and failure mode. Treating them through isolated feature definitions and dataset-specific workflows limits reproducibility and cross-study comparison. A deployment-consistent alternative requires explicit feature schemas, fusion rules, and validation protocols that remain coherent across modalities and data sources [12].

Portable hardware also imposes firm limits on local computation, memory, bandwidth, and energy use. Continuous transmission of raw biosignals is often unsuitable under intermittent connectivity and constrained operating budgets [13,14]. Consequently, time-bounded operations such as segmentation, compact feature extraction, and lightweight inference are better placed at the edge, whereas training, cross-dataset analysis, interpretability, aggregation, and model lifecycle management are more appropriately assigned to cloud resources. Edge–cloud asymmetry, therefore, shapes the analytical design itself, rather than serving as a later implementation optimisation. This shift from sensor-centric design toward deployment-aware analytics forms the background for the framework developed in the present study.

### 1.2. Motivation for Multimodal Physiological Analytics

Despite the growing sophistication of machine-learning pipelines for portable and wearable biosensor systems, many portable biosignal pipelines continue to rely primarily on single-modality EEG or EMG inference [15]. Although unimodal systems reduce sensing and computational complexity, they remain vulnerable to motion artefacts, electrode displacement, physiological variability, and environmental interference [16]. Recent wearable-stress biosensor reviews emphasise that real-world stress inference is constrained jointly by biomarker choice, sensing form factor, and continuous-readout feasibility rather than model design alone. This strengthens the case for deployment-aware multimodal analytics rather than single-signal optimisation [17].

Multimodal analytics can improve robustness by combining signals with different physiological origins and failure characteristics. Complementary channels may provide redundancy, contextual stabilisation, and greater tolerance to partial signal degradation. These benefits can often be obtained through feature-level fusion without requiring high-dimensional end-to-end architectures that exceed the computational envelope of embedded hardware [18].

The broader adoption of multimodal analytics in portable biosensor systems has nevertheless been slowed by structural fragmentation across the current research landscape. Public biosignal repositories are often modality-specific, task-confined, or evaluated within narrow methodological silos, and multimodal fusion strategies are commonly reported in isolation rather than across systematically aligned application domains. This lack of structured alignment between dataset characteristics, analytical objectives, and deployment constraints limits cross-study comparability and weakens system-level scalability, particularly when methods developed for offline experimentation are carried toward continuous, deployment-constrained inference [19,20]. In wearable-facing research, this fragmentation is especially problematic because low-power operation, partial signal loss, and heterogeneous sensing conditions impose tighter demands on analytical coherence than those encountered in static laboratory workflows. The present study addresses this problem through a dataset-to-application mapping and a hybrid edge–cloud pipeline governed by a unified feature and deployment contract.

### 1.3. Research Objectives and Scope

This study completes the analytical translation of the previously reported RCG architecture. The companion study established the sensing, communication, and storage platform; the present work defines the organisation, evaluation, adaptation, and deployment of its machine-learning workflow.

The study makes four contributions. First, it maps representative public biosignal repositories to physiological monitoring domains in order to expose modality-coverage gaps and dataset fragmentation. Second, it develops an asymmetric edge–cloud pipeline in which segmentation, schema-constrained feature generation, and lightweight inference are assigned to the edge, while training, adaptation, interpretability analysis, and longitudinal aggregation are assigned to the cloud. Third, it formalises a public-to-hardware adaptation pathway through a shared 13-feature intersection, followed by extension to the complete 17-feature hardware schema. Fourth, there is evaluation of repositories comprising WESAD [21], DEAP [22], PAMAP2 [23], EMGGestures [24], and EEGMMIDB [25] under subject-disjoint protocols, together with session-disjoint evaluation across five bench-acquired hardware sessions.

The scope of the study is intentionally narrow. Hardware characterisation, sensor integration, communication protocols, and power-management considerations are addressed in the accompanying architecture-focused study and are therefore not revisited here [5]. Likewise, the present paper does not address clinical diagnosis, therapeutic intervention, or patient-specific decision support. All evaluations are limited to analytics-oriented feasibility assessment: the public branch uses five publicly available repositories under subject-disjoint GroupKFold protocols, and the hardware branch uses five bench-acquired sessions under session-disjoint GroupKFold; no new human-subject data are collected, and no ethical approval is required within the present study design. This constrained scope is adopted deliberately so that the work can offer a reproducible analytical framework for portable multimodal biosensor systems without overstating translational or clinical readiness. The analytical boundary of the present work within the broader RCG stack is summarised earlier in Figure 1.

The remainder of this paper is organised as follows. Section 2 reviews the relevant literature on machine learning for non-invasive biosensor systems, multimodal physiological analytics, edge inference, and hybrid edge–cloud processing, and introduces the dataset-to-application coverage analysis that motivates the proposed framework. Section 3 formalises the data flow and machine-learning problem setting, including the rationale for asymmetric edge–cloud partitioning. Section 4 describes the analytical architecture, covering feature design, model construction, multimodal fusion, and the three-stage domain-adaptive training strategy. Section 5 presents feasibility-oriented evaluations across WESAD, DEAP, PAMAP2, EMGGestures, and EEGMMIDB under subject-disjoint validation. Section 6 discusses the findings, limitations, and implications for portable deployment-aware biosignal analytics. Section 7 concludes the paper.

## 2. Related Work

Research on machine learning for portable and wearable biosensor systems has developed along several partially overlapping trajectories, including non-invasive physiological signal analysis, multimodal state inference, resource-constrained edge deployment, and cloud-supported analytical orchestration. Although each of these areas has advanced substantially, their methodological development has often remained fragmented, with improvements in sensing modality, model design, and deployment architecture frequently evaluated within isolated experimental settings rather than as components of a unified analytical pipeline. Consequently, the interaction between multimodal biosignal integration, machine-learning workflow design, and deployment constraints remains unevenly addressed across the literature, particularly in portable and edge-limited contexts. Against this background, the present section reviews prior work across five connected themes: machine learning for non-invasive biosensor and BCI systems; edge and cloud computing in biosensor pipelines; lightweight and resource-constrained inference; hybrid edge–cloud analytical frameworks; and, finally, a structured synthesis of the resulting methodological gaps through dataset-to-application coverage analysis that motivates the framework developed in the remainder of this paper.

### 2.1. Machine Learning for Non-Invasive BCI Biosensors

Non-invasive biosensor platforms for physiological monitoring predominantly rely on electrophysiological sensing modalities that remain feasible under portable and wearable acquisition constraints, with EEG retaining a central position because of its accessibility and high temporal resolution [26]. Surface-recorded biosignals, however, are intrinsically low in signal-to-noise ratio, highly susceptible to artefacts, and strongly non-stationary across sessions and subjects, such that the resulting inference problem is fundamentally statistical rather than deterministic [27,28]. For this reason, the evolution of non-invasive BCI and biosensor analytics has remained closely coupled to advances in machine learning, particularly in workflows designed to transform high-variance temporal signals into stable intent- or state-level predictions under imperfect recording conditions.

A canonical machine-learning workflow for non-invasive biosignal analysis typically comprises preprocessing, segmentation into short analysis windows, feature extraction, and supervised classification. Within this structure, feature engineering has historically played a decisive role, with time-domain descriptors and spectral summaries used to preserve discriminative information while attenuating nuisance variation and acquisition noise. On top of such representations, a broad range of classifiers has been explored, including linear methods, margin-based models, probabilistic formulations, and ensemble learners, with model selection generally shaped by both the physiological task and the operational constraints of the target platform [29]. Inter-subject variability and session drift remain particularly important because high performance under record-wise or within-subject evaluation does not necessarily translate to unseen users or later sessions.

Deep learning reduces dependence on handcrafted descriptors by learning hierarchical representations directly from minimally processed signals. Such models can perform strongly when sufficient data and computational resources are available, but they may also require larger training sets, more complex optimisation, and additional safeguards against distributional shift [30,31]. Recent convolutional approaches have reported strong results in specific EEG classification settings; however, they are commonly evaluated under offline or workstation-class assumptions, and their transfer to latency-sensitive, resource-bounded portable inference remains non-trivial without retraining, compression, or architectural simplification [32]. Also, recent compact EEG deep-learning models have shown that explicit modelling of temporal dynamics and spatial asymmetry can materially improve affective EEG decoding. This is precisely the class of representation that the present compact, channel-averaged schema does not attempt to reproduce under its hardware-aligned constraints [33].

Taken together, prior work establishes that non-invasive biosensor analytics is fundamentally a machine-learning problem operating under persistent noise, non-stationarity, and user variability. Yet much of the literature continues to evaluate models within modality-specific and dataset-specific silos, with comparatively limited attention to deployment-aware workflow design or scalable integration across heterogeneous physiological streams. This unresolved gap motivates the present study, which prioritises structured machine-learning workflows that remain compatible with multimodal expansion and hybrid edge–cloud execution, rather than treating model complexity itself as the principal axis of progress.

### 2.2. Multimodal Physiological Analytics in Wearable Settings

Unimodal EEG- or EMG-centred pipelines simplify sensing and model construction but remain vulnerable to modality-specific degradation, including motion artefacts, electrode displacement, impedance variation, and physiological non-stationarity [34]. These limitations become more consequential in wearable-facing settings, where signal quality cannot be controlled continuously. Low-cost EEG systems further illustrate the trade-off between portability and channel richness, because compact form factors restrict the spatial information available to downstream models [35].

Multimodal analytics addresses this limitation by combining signals with different physiological origins, temporal characteristics, and failure modes. Neural, muscular, autonomic, and peripheral channels can provide complementary evidence, allowing one modality to contextualise or stabilise another when signal quality deteriorates [36,37]. Importantly, such gains do not necessarily require highly elaborate fusion architectures; in many settings, feature-level or decision-level integration is sufficient when modalities are selected and aligned according to physiological relevance rather than algorithmic convenience alone.

The principal value of multimodal fusion lies not merely in extracting marginal gains on idealised benchmark tasks, but in improving inference stability, contextual validity, and resistance to modality-specific corruption under realistic operating conditions. This is particularly important in wearable-oriented monitoring, where continuity, tolerance to imperfect acquisition, and analytical resilience are often more valuable than isolated peak accuracy under laboratory conditions.

Despite these advantages, the literature remains fragmented across modality combinations, annotation schemes, cohort structures, and validation protocols [38]. Results obtained on one repository, therefore, cannot be assumed to transfer directly to another, even when both are described as multimodal.

A further limitation is that many fusion studies assume centralised computation and sustained access to high-dimensional inputs. Such assumptions may conflict with the memory, latency, bandwidth, and energy constraints of portable systems [39]. These constraints point toward a more selective analytical design in which multimodal integration is organised around structured feature representations and fusion logic that remain viable within hybrid edge–cloud settings. It is precisely this need for deployment-aware multimodal analytics, rather than multimodality in abstraction, that motivates the framework developed in the present work.

### 2.3. Edge-Deployed and Resource-Constrained Machine Learning

The transition of BCI and biosensor analytics from laboratory-bound workflows to portable and wearable platforms introduces stringent constraints on computation, memory, energy consumption, and latency. Embedded biosignal inference must operate within bounded memory, computation, latency, and energy budgets [40,41]. This constraint is especially significant in wearable-facing biosensor systems intended for healthcare monitoring or human–machine interaction, where analytical responsiveness and low-overhead execution are not secondary implementation concerns, but conditions of practical usability.

Lightweight classifiers operating on structured time- and frequency-domain descriptors remain attractive because feature extraction and decision logic can be evaluated separately, parameter storage is modest, and runtime behaviour is comparatively predictable [42,43]. These properties are particularly important in BCI and wearable biosensor contexts, where inference latency directly influences system responsiveness, feedback timing, and the stability of downstream interaction or monitoring tasks.

Long-term adaptation introduces a separate challenge. Continuous on-device retraining is difficult to validate and may destabilise inference when local data are sparse or non-stationary. Hybrid architectures address this problem by separating fixed-parameter edge inference from upstream training, aggregation, and model revision [39]. Latency-sensitive segmentation, feature generation, and classification can remain local, whereas computationally intensive or episodic processes are assigned to the cloud.

These principles directly motivate the present study’s emphasis on lightweight, window-bounded models at the edge and cloud-assisted analytical support downstream, thereby preserving real-time responsiveness without abandoning scalability or reproducibility.

### 2.4. Dataset-to-Application Coverage Analysis

Dataset selection determines which physiological tasks and deployment assumptions can be evaluated meaningfully. Public repositories differ in modality composition, cohort size, annotation granularity, acquisition context, and class structure. Treating them as interchangeable benchmarks can therefore conflate model behaviour with dataset-specific properties. To address this limitation, the present work organises the five public repositories used in this study according to two linked criteria: the biosignal modalities available within each dataset, and the primary annotation targets that define the associated learning task. The included modalities span neural, neuromuscular, inertial, and peripheral physiological channels, whereas the annotation targets correspond to application-relevant outcomes such as motor intent, affective or stress-related state, gesture class, and movement context. This organisation allows each repository to be interpreted in relation to representative monitoring applications rather than evaluated in isolation. The resulting dataset-to-application alignment summary is presented in Table 1.

Table 1 shows that no single repository covers the complete application space. WESAD primarily supports physiological stress and affective-state analysis; DEAP provides affective EEG; PAMAP2 represents activity and movement context; EMGGestures supports neuromuscular gesture decoding [44]; and EEGMMIDB provides motor-imagery EEG [45,46]. These repositories are therefore complementary rather than interchangeable.

**Figure 2:**
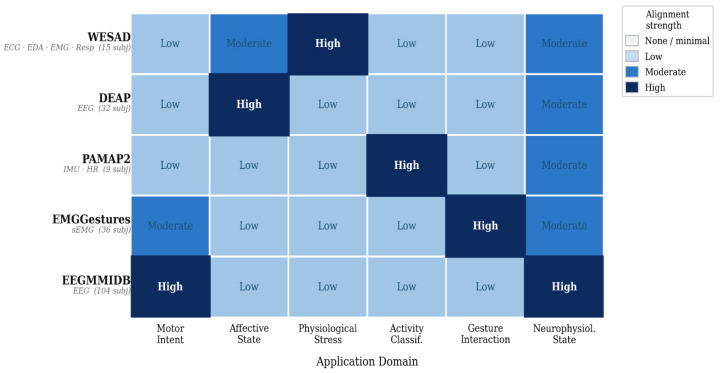
Dataset-to-application alignment heatmap for the five public biosignal repositories used in this study. Rows: WESAD, DEAP, PAMAP2, EMGGestures, and EEGMMIDB. Columns: motor intent recognition, affective-state monitoring, physiological stress assessment, activity classification, gesture-based interaction, and neurophysiological state analysis. Cell intensity reflects alignment strength according to modality composition, label granularity, and deployment relevance. Gaps indicate application domains with limited or no representative support across current public repositories.

Taken together, these repositories span four major biosignal domains, namely cortical motor intent, cortical affective state, multimodal physiological stress, and neuromuscular gesture, while mapping onto six canonical application areas: motor intent recognition, affective-state monitoring, physiological stress assessment, activity classification, gesture-based interaction, and broader neurophysiological state analysis. The corresponding coverage pattern is visualised in Figure 2.

Figure 2 highlights two limitations of the public-dataset landscape. First, modality and task coverage remain fragmented, with each repository representing only a subset of the intended application domains. Second, ambulatory, longitudinal, and deployment-facing scenarios remain weakly represented. The present study, therefore, preserves the native modalities and label spaces of each repository rather than imputing unavailable channels or forcing cross-dataset label equivalence. Recording context is likewise treated as an experimental constraint because models validated under controlled conditions may not reflect behaviour under portable or body-proximate operation [47]. The mapping developed here, therefore, serves both as a synthesis of the current literature landscape and as the design basis for the evaluation strategy reported in Section 5.

### 2.5. Hybrid Edge-Cloud Frameworks

Hybrid edge–cloud architectures separate latency-sensitive local processing from computationally intensive or longitudinal analytics. In portable biosensor systems, the edge supports responsive inference and local autonomy, whereas model training, cross-session aggregation, retrospective analysis, and model revision are more appropriately assigned to centralised infrastructure. Prior work in health IoT and cyber–physical systems has shown that this division can preserve local responsiveness while allowing data-intensive processing to be performed upstream [48]. Recent surveys of EEG-based BCI integration within connected industrial environments frame practical deployment as a distributed-systems problem involving latency, communication reliability, interoperability, security, and resource orchestration in addition to decoding performance. These requirements support an explicit separation of sensing, local inference, gateway transport, and cloud-side lifecycle management rather than a monolithic cloud-dependent pipeline [49]. In wearable-facing biosensor systems, this distinction is especially consequential because healthcare-monitoring and human-interaction use cases frequently require both immediate local interpretation and longer-horizon analytical support that exceed the stable operating envelope of the edge device itself.

Within such architectures, embedded nodes can remain inference-oriented while periodically receiving models derived from broader data accumulated across users, sessions, or operating conditions [50]. Cloud resources also support structured persistence, reproducible model development, comparative evaluation, and lifecycle management that are difficult to sustain on-device. Feature-centric upstream exchange, in which compact representations and inference outputs are transmitted instead of continuous raw biosignals, can further reduce bandwidth and persistence requirements while retaining sufficient information for downstream analysis [51]. For wearable-oriented systems, such compact upstream exchange is often preferable to sustained raw streaming, because it better respects the energy, bandwidth, and persistence constraints that accompany body-proximal continuous sensing.

Despite these advantages, many existing hybrid machine-learning frameworks remain described at a relatively high level of abstraction, with limited treatment of how biosignal-specific constraints, including window-bounded inference, modality heterogeneity, and non-stationarity, interact with practical pipeline design. In BCI and physiological monitoring systems, the absence of explicit coordination between edge-side inference and cloud-side analytics can lead to fragmented workflows, ambiguous component responsibilities, and weak continuity between offline model development and deployed inference. This weakness becomes especially visible when multimodal sensing, portable operation, and long-horizon evaluation must be addressed within the same analytical framework. The present study, therefore, approaches hybrid edge–cloud design not as a generic systems pattern, but as an explicitly biosignal-aware analytical strategy shaped by modality structure, deployment limits, and the requirements of reproducible physiological inference.

### 2.6. Summary and Identified Research Gaps

The reviewed literature establishes the value of machine learning for non-invasive biosignal interpretation, multimodal fusion for improving inference stability, lightweight models for constrained execution, and hybrid architectures for separating local responsiveness from cloud-side scalability. Nevertheless, these advances remain only partially integrated.

First, biosignal-learning studies remain fragmented across modalities, repositories, task definitions, and evaluation protocols. Reviews of wearable stress monitoring similarly report substantial protocol dependence and limited evidence of cross-dataset generalisation [52]. This fragmentation restricts cross-study comparison and makes it difficult to determine whether an analytical workflow remains coherent beyond the dataset for which it was developed.

Second, machine-learning design and deployment architecture are often treated independently. Classifiers may be optimised under centralised or workstation-class conditions, while embedded studies frequently report model execution without preserving continuity with the feature-generation, validation, and adaptation procedures used during development. The interaction among multimodal representation, grouped evaluation, resource constraints, and execution placement, therefore, remains insufficiently formalised.

Third, hybrid edge–cloud frameworks commonly assign broad responsibilities to each tier without defining a stable schema and model-lifecycle contract between them. Limited attention is given to how models are adapted, validated, exported, updated, or rolled back while maintaining compatibility with the deployed feature representation.

The present study addresses these gaps through a unified workflow combining heterogeneous public repositories, subject-disjoint evaluation, multimodal feature-level fusion, a shared public-to-hardware feature intersection, session-disjoint hardware validation, and schema-locked edge–cloud deployment. Section 3 formalises the resulting data flow, learning problem, feature contract, and computational partitioning strategy.

## 3. Data Flow and Problem Formulation

The analytical framework is defined by four connected design decisions: how the public and hardware data branches enter the pipeline, how heterogeneous learning tasks are formulated, how feature compatibility is maintained across training and deployment, and how computation is divided between edge and cloud tiers. Together, these decisions establish the representational and evaluation contract used throughout the study. Embedded classification is based exclusively on the four synchronous biosignal channels and the resulting 17-feature schema; auxiliary sensors within the broader platform do not participate in edge inference.

### 3.1. End-to-End Analytical Data Flow

The pipeline contains two input branches that converge within a shared training and deployment pathway. The public branch processes WESAD, DEAP, PAMAP2, EMGGestures, and EEGMMIDB using dataset-specific windowing and modality-aligned feature extraction. It supports unimodal evaluation, multimodal fusion, and extraction of public-domain statistics within the shared 13-feature intersection.

The hardware branch processes synchronous sEMG, EEG, ECG, and EOG signals acquired at 250 Hz. These signals are converted into the complete 17-feature RCG representation. In addition to the 13 features shared with the public branch, the hardware schema contains four deployment-specific variables: two ECG-derived HRV variability proxies, one EOG blink-rate feature, and one EEG quality indicator. The complete representation is used for hardware adaptation and edge inference. Throughout this study, multimodal fusion refers specifically to feature-level integration of the synchronous biosignal channels and does not include the auxiliary telemetry stream.

**Figure 3:**
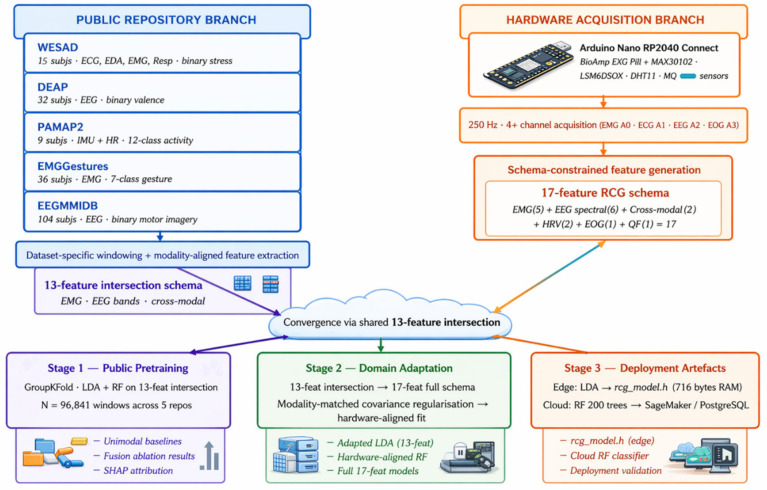
End-to-end analytical data flow of the machine-learning pipeline. The public branch comprises repository loading, dataset-specific windowing, and modality-aligned feature extraction. The hardware branch comprises 250 Hz four-channel acquisition followed by schema-constrained feature generation under the 17-feature representation. Both branches converge within a public-pretraining-to-hardware-adaptation training pathway that produces cloud-resident and edge-deployable inference artefacts.

The two branches converge through three analytical stages. Stage 1 develops public-domain models and modality-matched statistics within the 13-feature intersection. Stage 2 adapts this representation to the hardware distribution and extends it to the full 17-feature schema. Stage 3 produces a schema-locked edge artefact for embedded inference and a cloud-resident model for downstream analysis, retraining, and lifecycle management. The complete flow is shown in Figure 3.

As clarified by Figure 3, the public and hardware branches serve different evidential roles. The public repositories test whether the analytical workflow remains coherent across heterogeneous physiological tasks under subject-disjoint evaluation, whereas the hardware branch tests whether the same schema and deployment logic remain executable under session-disjoint bench conditions. Their connection through the shared 13-feature intersection provides representational continuity without treating public datasets as direct surrogates for the embedded rig. Stronger claims concerning ambulatory, multi-subject, or clinical utility require subsequent on-body validation and are outside the present evaluation scope.

### 3.2. Problem Formulation Across Heterogeneous Biosignal Repositories

The learning problem is formulated uniformly across all data sources as supervised classification at the window level. Each temporal window yields a fixed-dimensional feature vector and is assigned a label drawn from the annotation scheme native to the source dataset. Label spaces are therefore treated as intrinsic properties of the individual repositories, spanning binary formulations for stress detection and motor imagery, multi-class settings for activity and gesture recognition, and task-specific affective stratifications where applicable. No attempt is made to merge or harmonise these label spaces across repositories. This design preserves the integrity of the native annotation structure of each dataset and avoids artificial alignment that would obscure genuine differences in task definition, label semantics, and classification difficulty.

Modality availability is treated as an experimental constraint rather than as a deficiency requiring correction. Because the selected repositories differ substantially in their available physiological channels, no missing modalities are provided, and no absent channels are synthesised. Unimodal baselines are therefore constructed only from modalities natively present within a given repository, whereas multimodal configurations are restricted to those channels that are actually available in that dataset. This constraint ensures that observed performance differences reflect genuine effects of modality integration rather than artefacts introduced by data-completion strategies [8].

To reduce optimistic leakage across temporally correlated biosignal samples, all train-validation partitions in the public benchmark branch are enforced as subject-disjoint. Cross-validation is implemented through grouped folds in which all windows from a given subject are assigned exclusively to either the training or the validation partition, and never split across both. Window-level random partitioning is explicitly excluded because it inflates performance estimates by allowing models to encounter samples from the same subject during both fitting and evaluation [20]. This subject-disjoint protocol is applied uniformly across all five public repositories and serves as the primary evaluation rule for the benchmark arm of the analytical pipeline.

For the hardware branch, the same anti-leakage principle is applied directly. The hardware dataset comprises five bench sessions with distinct signal-level characteristics. Session identity is used as the grouping variable in a leave-one-session-out GroupKFold protocol (k = 5), so that all windows from a given session appear exclusively in either the training or the validation partition. This session-disjoint structure prevents leakage across temporally correlated windows while providing a cross-session robustness estimate that is structurally analogous to the subject-disjoint protocol applied in the public benchmark branch.

### 3.3. Feature Representation and Schema Constraint

Feature extraction is applied to each temporal analysis window to produce a fixed-dimensional representation that remains compatible with both cloud-side model development and edge-oriented inference. The pipeline adopts a fixed 17-feature schema organised across five physiological groups: five EMG time-domain descriptors capturing amplitude and activation dynamics; six EEG spectral features spanning canonical frequency bands together with spectral entropy; two cross-modal spectral descriptors; two HRV-derived features from ECG; and one EOG-derived feature, supplemented by a signal-quality indicator to complete the 17-dimensional representation. The EEG signal-quality indicator is computed per analysis window: the raw EEG samples are mean-centred and their variance is calculated in ADC^2^ units; a binary value of 1 is assigned when this variance exceeds a threshold of 30 ADC^2^, and 0 otherwise. The threshold was established from hardware acquisition logs to separate windows near the observed noise floor from those containing measurable signal variation. This indicator does not detect motion artefacts or confirm physiological EEG authenticity—high variance can arise from artefact sources as well as genuine neural activity—and its role is to provide the classifier with a simple per-window signal-variation flag as one of the 17 input features. All features are computed through standardised time- and frequency-domain operations, with spectral estimates derived using Welch-based procedures [43] and temporal descriptors selected for low-overhead physiological interpretability [47]. This design favours analytical stability and deployment continuity over dataset-specific feature proliferation.

A central constraint of this representation is the distinction between the full 17-feature hardware-aligned schema and the reduced 13-feature intersection available across the public repositories used for model development. The hardware-exclusive subset comprises the HRV-derived pair, the EOG blink-rate feature, and the signal-quality indicator, none of which has a direct and consistently aligned counterpart across the selected public datasets. The shared 13-feature intersection, consisting of the EMG, EEG-band, and cross-modal groups, therefore functions as the representational contract linking public-data pretraining to hardware-facing deployment. This explicit schema partition avoids artificial feature imputation and preserves continuity across training stages while acknowledging genuine differences in modality availability.

Before model fitting, feature vectors are standardised using scaling parameters estimated exclusively from the training partition. This prevents information leakage while preserving comparability across data sources and evaluation folds. Feature dimensionality and ordering are enforced through schema validation at each pipeline stage, ensuring that cloud-side training, transfer-oriented adaptation, model export, and edge-facing inference all operate under the same representational assumptions regardless of data origin. In this sense, the schema functions not merely as a feature inventory, but as the formal mechanism through which deployment consistency is maintained across the analytical workflow.

### 3.4. Asymmetric Edge–Cloud Partitioning Strategy

The analytics pipeline assigns computational responsibility across edge and cloud tiers through an explicitly asymmetric design. Machine-learning operations are not distributed uniformly; instead, each stage is placed according to its latency sensitivity, data dependency, and computational footprint. The edge tier is reserved for time-bounded operations, namely windowed segmentation, schema-constrained feature generation, and lightweight fixed-parameter inference, all of which must execute within deterministic resource budgets and without dependence on continuous network availability [53]. The cloud tier, by contrast, is assigned episodic and data-intensive operations, including supervised model training, cross-dataset adaptation, longitudinal aggregation, and model versioning, which benefit from centralised compute and accumulated data but do not impose strict real-time constraints [51].

This asymmetry is methodological rather than merely architectural. Edge-side inference is stateless with respect to learning: model parameters are fixed at deployment and remain unchanged during routine operation. Model revision occurs upstream and is propagated downstream only through episodic update cycles, thereby decoupling inference stability from training dynamics. Such separation is well motivated in portable biosensor settings, where intermittent connectivity, limited memory, and constrained energy budgets make continuous on-device learning both impractical and difficult to validate reliably [18,42]. Data exchange between tiers is correspondingly restricted and structured. Derived feature representations and inference outputs are eligible for upstream transfer, whereas model updates move downstream from cloud to edge. Continuous raw-signal transmission is therefore avoided, preserving a clear analytical interface between the two computational tiers.

Within the framework, this partition maps directly onto the modular boundary established in the companion architecture: the Cortex layer supports edge-side feature generation and inference, whereas the Vault layer manages cloud-side training, model persistence, and analytical aggregation. The partition is therefore not a secondary implementation convenience, but the governing principle through which real-time responsiveness at the edge is made compatible with broader analytical scalability upstream. Section 4 describes how this division is realised within the machine-learning architecture developed in the present study.

### 3.5. Deployment-Aware Evaluation Logic

Evaluation within the analytics pipeline is designed to reflect deployment conditions rather than to maximise benchmark performance under assumptions that would not hold in practice. Two constraints govern all experimental protocols reported in Section 5.

The first constraint is subject-disjoint partitioning. All cross-validation folds in the public benchmark branch are constructed such that every window from a given subject appears exclusively in either the training or the validation set, and never in both. This is enforced using grouped cross-validation with subject identifiers as the grouping variable, applied uniformly across all five public repositories. Window-level random partitioning is explicitly excluded because it allows the model to encounter temporally correlated samples from the same subject during both fitting and evaluation, thereby inflating performance estimates that do not generalise to unseen individuals. Wearable time-series studies have explicitly shown that leave-one-subject-out validation is the appropriate model-selection regime when the deployment target is an unseen user, because subject leakage otherwise inflates apparent performance. The same anti-leakage rationale underlies the grouped folds used throughout the public branch here [54].

The second constraint is deployment-consistent feature generation. Feature extraction parameters, including window length, stride, and schema dimensionality, are held fixed within each analytical branch so that the representations used during cloud-side training remain structurally compatible with those produced during hardware-side inference. This preserves the schema contract established in Section 3.3 and ensures that model evaluation reflects the same representational assumptions that govern deployment. Reported metrics are therefore interpretable as estimates of deployment-ready analytical behaviour, within the limits of bench-level validation, rather than as results obtained under a disconnected offline-processing regime.

Taken together, these constraints define the evaluation logic of the present study as deployment-consistent rather than benchmark-optimised. That distinction is central to the analytical claims made in Section 5 and to the limitations discussed later in Section 6.

## 4. Analytical Architecture and Model Development

This section instantiates the formal framework defined in Section 3 as a concrete multi-stage analytical pipeline. It shows how the data-flow structure, schema constraints, asymmetric edge–cloud partitioning, and deployment-consistent evaluation logic are realised within the model-development workflow of the system. The presentation follows the operational order of the pipeline: public-dataset processing and baseline construction, multimodal fusion and ablation analysis, joint training and cross-domain adaptation, and, finally, deployment artefact generation for edge and cloud targets. The emphasis throughout is placed on functional role, representational continuity, and deployment compatibility rather than on algorithmic novelty in isolation. Where individual model classes are introduced, their selection is governed by the constraints formalised in Section 3.4, namely determinism, bounded resource footprint, and compatibility with the fixed feature schema.

### 4.1. Public Dataset Processing and Baseline Model Construction

Each public repository is processed through a dataset-specific loading and segmentation procedure that preserves the native sampling characteristics, annotation structure, and physiological task definition of the source dataset. Windowing parameters are selected to balance temporal responsiveness, feature stability, and downstream deployment relevance while remaining compatible with the unified analytical workflow of the pipeline. Across all repositories, feature extraction is performed within the shared 13-feature intersection space defined in Section 3.3, thereby preserving continuity between public-domain analysis and the later hardware-aligned adaptation branch. The dataset-specific processing configuration used throughout the public benchmark stage is summarised in Table 2.

Table 2 makes explicit that deployment consistency in the present study is enforced not by imposing identical raw-signal conditions across repositories, which would be impossible, but by standardising the analytical contract under which each dataset enters the pipeline. Windowing remains dataset-specific because the underlying acquisition regimes and task structures differ, yet the evaluation rule, feature-space logic, and subject-disjoint protocol are kept aligned. This balance allows repository-native structure to be preserved without breaking continuity across the broader analytical workflow.

Unimodal baselines are then constructed for each dataset and for each natively available modality using three classifier families chosen for complementary behaviour under deployment-relevant constraints. LDA is included because of its closed-form training and minimal inference burden [15]. SVM with an RBF kernel provides a compact non-linear margin-based baseline under fixed feature representations [55]. Random Forest is included as an interpretable ensemble learner capable of modelling non-linear decision structure without requiring representation learning [56]. All models operate on standardised feature vectors and are evaluated under the subject-disjoint grouped cross-validation protocol defined in Section 3.5, with five folds applied consistently across the public repositories. Performance is reported as mean ± standard deviation across folds using accuracy and macro-F1, thereby capturing both aggregate classification performance and class-balanced behaviour under unequal label distributions.

Regarding model configuration, unless otherwise stated, all public-branch classifiers were evaluated under fixed, non-nested settings to preserve comparability across repositories. LDA used the default closed-form formulation. SVM employed an RBF kernel with C = 1.0 and gamma = scale. Random Forest used n_estimators = 200 and max_depth = 15, with random_state = 42 and parallel fitting enabled where supported. In the transfer branch, the public Random Forest pretraining stage used 150 trees, followed by warm-start expansion with 50 additional hardware-trained trees. For deployment comparison, the cloud-resident Random Forest retained the 200-tree, depth-15 configuration, whereas the edge fallback Random Forest was pruned to 20 trees with a maximum depth of 5 to remain compatible with the embedded memory budget during deployment comparison. Within the analytical workflow, these unimodal results serve two roles. First, they establish dataset- and modality-specific reference points against which subsequent multimodal fusion results can be interpreted. Second, they provide the public-domain baseline layer required for later transfer-oriented training and deployment-aware model selection. The unimodal stage is therefore not treated as an isolated benchmarking exercise, but as the reference frame from which the broader analytical architecture developed in the following subsections proceeds.

### 4.2. Multimodal Fusion and Ablation Analysis

For repositories containing multiple natively available modalities, a structured ablation procedure is used to characterise the contribution of individual modalities and modality combinations to classification performance. Evaluation begins with unimodal configurations, in which the feature space is restricted to a single physiological source for each run. Multimodal configurations are then constructed through feature-level fusion, whereby feature vectors extracted independently from each available modality are concatenated within a shared analysis window before model fitting and inference. This strategy preserves modality-specific preprocessing while retaining a common workflow structure across unimodal and multimodal branches, thereby remaining compatible with the deployment-oriented analytical logic established in Section 3.4. For repositories exposing only a single modality, ablation is restricted to within-modality analytical comparisons rather than cross-modality fusion.

Feature-level fusion is preferred over signal-level and decision-level alternatives for two reasons. First, it preserves the representational logic of the overall pipeline by allowing each modality to undergo preprocessing and feature extraction in its native form before integration. Second, it supports direct post hoc assessment of modality contribution without introducing fusion-specific architectural parameters that would complicate interpretation under a fixed deployment-oriented workflow [12]. Attribution analysis is performed on the Random Forest branch using SHAP-based feature importance where supported, with permutation importance used for the hardware-aligned feature ranking reported later in the pipeline. Feature contributions are examined both individually and after aggregation by modality group, allowing the relative influence of neural, muscular, autonomic, and contextual signals to be interpreted across datasets and task settings. Recent multimodal headset studies likewise report that synchronised fusion across neural and peripheral channels is useful when modalities are aligned under a common temporal support. This supports the present paper’s preference for structured feature-level fusion rather than treating each modality as an isolated analytical stream [57].

The resulting ablation design yields, for each applicable repository, a structured comparison spanning unimodal baselines, pairwise multimodal combinations where relevant, and full multimodal configurations. Performance deltas between unimodal and fused settings are then computed under the subject-disjoint evaluation protocol defined in Section 3.5, allowing the marginal contribution of multimodal integration to be quantified without collapsing differences in task structure or dataset composition. These outputs provide the principal analytical basis for the multimodal feasibility interpretation presented in Section 5.

### 4.3. Joint Training and Cross-Domain Adaptation

The joint training stage links public-domain model development to hardware-aligned deployment through a structured staged adaptation procedure. This design addresses a central representational asymmetry within the pipeline. The five public repositories provide broad supervised coverage across heterogeneous physiological domains, but only within the shared public-branch feature space aligned to the 13-feature intersection contract. The hardware branch, by contrast, provides the full 17-feature representation, yet over a substantially smaller labelled sample and under a narrower acquisition context. Training solely on hardware data from random initialisation would discard distributional information available across the public repositories, whereas direct transfer without adaptation would ignore the genuine shift between repository-derived features and the hardware-facing signal distribution [58]. The staged procedure is therefore designed to preserve transferable structure while explicitly adapting it to deployment-facing data.

In Stage 1, modality-matched public repositories are used to estimate within-class covariance structure within the shared feature blocks under the subject-disjoint evaluation logic defined in Section 3.5. The label spaces remain local to their source datasets and are neither pooled nor mapped onto the hardware task. This stage, therefore, supplies modality-specific covariance priors and public-domain reference statistics rather than transferable class centroids, class semantics, or decision boundaries. LDA and Random Forest are retained for different analytical purposes in the subsequent stages: LDA supports explicit covariance regularisation, whereas the Random Forest branch provides a schema-consistent non-linear comparison under the recognised class-space limitation.

Stage 2 performs hardware-aligned adaptation within the same 13-feature intersection space before extending the representation to the full 17-feature schema. For LDA, adaptation is implemented through regularisation of the hardware covariance matrix using modality-matched public covariance priors. Hardware class means, class priors, scaling parameters, and target labels are estimated exclusively from the hardware sessions; no class-semantic information, class centroids, or label assignments cross the domain boundary. For Random Forest, the incompatible public and hardware class spaces prevent principled transfer of class-specific tree votes or decision structure. The hardware RF branch is therefore fitted independently under the same schema and retained as a schema-consistent comparison, with its equality to scratch training reported as a null transfer outcome. The second adaptation step introduces the four hardware-exclusive variables, namely the two HRV descriptors, the EOG blink-rate feature, and the signal-quality indicator, thereby extending the hardware-aligned model from the shared 13-feature intersection to the full 17-feature deployment schema.

For LDA, the transition into the 17-feature space carries the selected covariance-regularisation setting into a fresh model estimated on hardware-aligned data under the complete schema. For Random Forest, the corresponding 17-feature model is estimated directly from the hardware sessions and is interpreted as a schema-consistent hardware fit rather than as a transferred public decision structure. This staged design preserves representational continuity between the public and hardware branches while ensuring that all target-task discrimination remains anchored to the hardware distribution. The resulting design preserves continuity across model-development phases without relying on artificial feature imputation or assuming direct equivalence between public and hardware distributions.

### 4.4. Edge and Cloud Deployment Artefact Generation

Following the joint training and adaptation stages, the pipeline produces two distinct deployment artefacts that operate under the same schema contract while targeting different computational tiers. The cloud-resident artefact is a Random Forest classifier trained on the full 17-feature hardware-aligned representation and retained for longitudinal analytics, comparative evaluation, and periodic retraining. The edge-resident artefact is an LDA classifier selected as the primary embedded inference engine because of its closed-form parameterisation, low execution overhead, and minimal memory footprint under the deployment constraints. A controlled comparison with alternative lightweight architectures, such as quantised 1D convolutional networks, was not conducted; such an evaluation would require a separately designed signal-tensor input pipeline, independent quantisation procedure, and deployment profiling on the same target hardware, and is identified as a future evaluation direction. This model is serialised into a schema-locked static representation suitable for direct inclusion in firmware, so that the trained decision boundary can be evaluated on each incoming feature vector through fixed coefficient operations without requiring dynamic memory allocation or architecture-specific learning logic at runtime.

Table 3 summarises the measured and estimated deployment profile of the edge LDA artefact on the target hardware, an Arduino Nano RP2040 Connect (model ABX00053; Arduino S.r.l., Monza, Italy), featuring a dual-core RP2040 Arm Cortex-M0+ processor operating at 133 MHz. Inference latency was measured directly on the target device via firmware micros() instrumentation over N = 500 inference cycles following a 50-cycle warmup, using a fixed deterministic input vector.

A full system-level power budget is not reported; the Nano, analogue front ends, storage, display, and LTE gateway have distinct power contributions and are not operated as a single battery-integrated wearable in the present bench configuration. The low inference latency SD (0.9 µs; CV < 1%) is consistent with the fixed-operation structure of the deployed LDA implementation, which involves a deterministic matrix multiply and argmax with no data-dependent branching. At the operational duty cycle of one inference per 5 s acquisition window, inference computation constitutes approximately 0.0022% of the total window duration. Prior to deployment, both artefacts undergo a validation step that verifies schema conformity, parameter integrity, numerical stability, and execution feasibility under the intended operating conditions. Models that fail to satisfy predefined latency or memory constraints are excluded from deployment irrespective of their offline performance, thereby enforcing a strict separation between analytical optimisation and operational readiness. Each retained artefact is associated with a specific training configuration, schema definition, and validation outcome, allowing model provenance to remain reproducible across update cycles and enabling systematic comparison between successive deployment versions [59].

Beyond the inference and model management pathway, the hardware branch generates a structurally separate environmental and physiological context stream through the auxiliary sensor suite. This telemetry stream—comprising gas sensor readings (MQ-3, MQ-7, MQ-9, MQ-135), ambient climate measurements (DHT11 temperature and relative humidity), photoplethysmographic heart rate and SpO_2_ (MAX30102), and skin temperature (MAX30205)—is sampled at 1 Hz and transmitted to the Vault layer through the same gateway uplink as the biosignal feature stream. It does not enter the 17-feature inference schema and is not used for window-level classification. Its analytical role operates at the session level in the cloud: post hoc window quality annotation, environment-to-inference correlation, session drift detection, and alert provenance tracing. Concretely, gas alarm flags and temperature anomalies recorded in the telemetry log are joined to the window-level inference record by timestamp, allowing the cloud to suppress or contextualise windows whose acquisition environment was compromised without modifying the edge inference logic or the schema contract. This design realises the longitudinal aggregation and retrospective analysis capabilities assigned to the Vault layer as concrete, operationally grounded functions rather than abstract architectural commitments.

Model revision is performed episodically rather than continuously. Updated artefacts are generated through cloud-side retraining on accumulated or revised data, validated against the incumbent baseline, and propagated to the edge tier only after passing the deployment gate described above. During the interval between updates, the edge system continues to operate using the previously validated model, thereby preserving uninterrupted inference without undefined intermediate states. Edge-resident inference remains fully functional in the absence of network connectivity, with cloud interaction restricted to asynchronous upstream transfer of derived feature summaries and downstream receipt of validated model updates. This offline-capable design follows directly from the asymmetric partitioning strategy defined in Section 3.4 and preserves analytical continuity under the intermittent connectivity conditions that commonly characterise portable biosensor deployment.

### 4.5. Operational Summary of the ML Stack

The four stages described in this section together form a unified and sequentially dependent analytical pipeline, in which the output of each stage serves as an input, constraint, or deployment condition for the next. Public-dataset processing and baseline construction establish the reference performance layer for subsequent fusion and adaptation analysis. Multimodal fusion and ablation analysis extend this reference layer by characterising modality interaction effects and producing the attribution outputs examined later in Section 5. Joint training and cross-domain adaptation then combine public-domain structure with hardware-aligned feature representations to produce schema-consistent models in both the shared 13-feature intersection space and the full 17-feature deployment space. Finally, deployment artefact generation transforms these adapted models into tier-specific outputs that operationalise the asymmetric partitioning strategy defined in Section 3.4. The functional responsibility of each stage, together with its active schema, computational tier, and principal outputs, is summarised in Table 4.

Table 4 shows that the stack is not a set of independent analytical modules, but a staged workflow in which representational continuity is preserved from public-domain modelling through to deployment-facing inference. In particular, the progression from the 13-feature intersection to the full 17-feature schema makes explicit where public-domain compatibility ends and hardware-specific deployment begins. This operational summary also clarifies why later results in Section 5 must be interpreted stage-wise rather than as a single undifferentiated benchmark output. Section 5 evaluates the outputs of Section 4.1 and Section 4.2 through unimodal baselines, multimodal fusion comparisons, cross-dataset performance patterns, and feature-level attribution, and also reports the deployment-oriented outcomes produced by Section 4.3 and Section 4.4.

## 5. Experimental Validation and Results

This section evaluates the proposed machine-learning architecture across the five public biosignal repositories selected through the dataset-to-application mapping strategy defined earlier, together with the hardware-aligned branch used for deployment-oriented feasibility assessment. The purpose of these experiments is not to claim state-of-the-art performance on any single benchmark, but to assess the feasibility, robustness, and interpretability of the analytics pipeline under unimodal, multimodal, and deployment-facing conditions. All evaluations are conducted using fixed windowing, feature extraction, schema validation, and model configurations consistent with the analytical constraints formalised in Section 3 and Section 4.

The reported results are organised to reflect progressive analytical depth across the pipeline. The section begins with unimodal baselines, which establish dataset- and modality-specific reference points under subject-disjoint evaluation. It then examines multimodal fusion and ablation behaviour under matched analytical conditions, followed by cross-dataset comparative interpretation of performance patterns under differing modality structures and task demands. The joint-training and hardware-adaptation stages are then evaluated as deployment-oriented feasibility steps rather than as generic transfer-learning benchmarks, after which the edge and cloud deployment artefacts are compared under the resource and schema constraints of the target system.

Explainability results are presented separately so that predictive performance is not conflated with interpretability claims. Feature- and modality-level attribution patterns are analysed to assess whether the observed decision behaviour remains consistent with the physiological and structural roles of the contributing modalities across datasets and task settings. Taken together, these experiments provide a structured evaluation of how multimodal learning, staged adaptation, asymmetric edge–cloud partitioning, and explainable analytics interact within the framework.

### 5.1. Datasets and Experimental Setup

This study reports feasibility-oriented experiments using five public biosignal repositories selected to span analytically distinct but deployment-relevant domains, namely multimodal physiological stress monitoring, cortical affective-state inference, inertial activity recognition, neuromuscular gesture decoding, and cortical motor-imagery classification. The dataset-to-application alignment established earlier in Table 1 provides the application-facing rationale for this selection, whereas the dataset-specific processing and split protocol defined in Table 2 fixes the methodological conditions under which all benchmark experiments are conducted. Together, these two layers ensure that the reported results are interpretable both in relation to their intended monitoring use cases and in relation to the deployment-consistent evaluation constraints of the pipeline.

The system evaluates unimodal and multimodal feature-level learning across WESAD, DEAP, PAMAP2, EMGGestures, and EEGMMIDB. WESAD provides multimodal physiological recordings and serves as the reference dataset for stress-oriented multimodal sensing. DEAP is used as the EEG-based benchmark for affective-state analysis under binary valence stratification. PAMAP2 represents inertial activity recognition through multimodal motion and heart-rate streams. EMGGestures provides the neuromuscular benchmark for gesture classification from surface EMG. EEGMMIDB provides the cortical benchmark for motor-imagery classification at scale. Collectively, these repositories define the final public-dataset basis for assessing the analytics pipeline under heterogeneous modality structure, task definition, and deployment relevance.

Task definitions are preserved strictly at the native label granularity of each dataset. WESAD is evaluated as a binary stress-versus-non-stress problem. DEAP is evaluated as a binary valence-classification task using a per-subject median split on the valence dimension. PAMAP2 is treated as a multi-class activity-recognition problem. EMGGestures is evaluated as a seven-class gesture-classification task. EEGMMIDB is evaluated in a binary motor-imagery formulation. No cross-dataset label harmonisation is imposed at this stage, because the purpose of the public benchmark branch is to characterise model behaviour within each repository’s own annotation structure before any later adaptation to the hardware-aligned branch.

All public-dataset train-validation partitions are enforced as subject-disjoint, because window-level random splits artificially inflate performance in time-series biosignals by allowing temporally correlated samples from the same individual to appear in both fitting and evaluation. This anti-leakage rule is applied uniformly across all five repositories and forms the primary evaluation constraint of the benchmark branch. Feature extraction is restricted to the shared 13-feature intersection space, and all models are trained and evaluated under the same deployment-consistent schema logic defined earlier. Accordingly, the metrics reported in the following subsections should be read not as isolated benchmark scores, but as stage-specific indicators of how the analytical architecture behaves under fixed representational and evaluation constraints.

### 5.2. Evaluation Metrics

Model performance is assessed using accuracy and macro-F1 as the primary summary metrics throughout the public benchmark branch. Accuracy is retained to indicate aggregate classification correctness under the native label structure of each dataset, whereas macro-F1 is used as the principal comparative metric because it weights all classes equally and is therefore more informative under unequal class distributions and heterogeneous task formulations [60]. This distinction is especially relevant in the present study, where the evaluated repositories span both binary and multi-class problems with markedly different class balances and baseline difficulties.

To support more detailed interpretation where appropriate, confusion analysis is used to characterise class-specific error structure, and receiver operating characteristic and precision–recall analysis are reported selectively for settings in which threshold behaviour and decision separation are analytically informative [61]. These supplementary views are not treated as substitutes for the primary summary metrics, but as diagnostic tools that clarify whether observed performance changes arise from broad class separation, skewed decision behaviour, or mode-specific confusion patterns under multimodal fusion. For this reason, they are introduced only in the result subsections where the additional interpretive value is justified by the task structure and model behaviour.

For hardware-aligned adaptation and deployment comparisons, the same accuracy and macro-F1 framework is retained so that public-domain, transfer-stage, and deployment-stage outputs remain directly comparable at the level of supervised classification behaviour. However, deployment-oriented interpretation extends beyond predictive metrics alone. For edge-facing artefacts, memory footprint and representational compactness are treated as co-equal evaluation dimensions because the practical viability of an embedded model depends not only on predictive performance, but on whether it satisfies the resource envelope of the target platform. Accordingly, later deployment comparisons are interpreted jointly in terms of classification behaviour and execution suitability rather than benchmark performance in isolation.

All reported values are expressed as mean ± standard deviation across evaluation folds where fold-based validation is applicable. For the public repositories, these summaries derive from subject-disjoint GroupKFold evaluation. For the hardware branch, values derive from session-disjoint GroupKFold evaluation (k = 5, leave-one-session-out) across five bench sessions, each with distinct signal characteristics. Both branches, therefore, apply the same anti-leakage principle—no windows from a given subject or session appear in both fitting and evaluation partitions—and the resulting metrics are directly comparable in their structural interpretation, differing only in whether the grouping variable is subject identity or session identity.

### 5.3. Unimodal Classification Performance

Unimodal evaluation establishes the reference performance layer for the analytics pipeline by quantifying how each dataset-modality pair behaves in isolation under the deployment-consistent feature schema and subject-disjoint validation logic defined earlier. Across the five public repositories, the strongest unimodal performance was observed for sEMG- and IMU-dominant tasks, whereas EEG-only tasks remained substantially weaker under the fixed, channel-averaged representation used throughout the present study. This separation is analytically important because it indicates that unimodal behaviour is governed not only by classifier family, but by the degree to which task-relevant structure is preserved by the constrained feature schema. The full unimodal results are summarised in Table 5.

Table 5 shows a clear separation between modality families. The strongest unimodal results arise in sEMG- and IMU-dominant settings, with EMGGestures and PAMAP2 providing the most stable class-balanced performance across models. By contrast, EEG-only tasks remain in the modest or near-chance regime under the deployment-consistent feature representation adopted here. This pattern indicates that the constrained feature schema preserves discriminative structure more effectively for amplitude-dominant neuromuscular and inertial tasks than for tasks that depend more heavily on richer spatial EEG structure [15,62].

Selected published benchmarks on datasets used in this study are provided for contextual orientation. Direct numerical comparison with results in Table 5 is not appropriate: published methods use dataset-native feature representations and task-specific tuning, whereas the present framework applies a fixed 13-feature cross-dataset schema under subject-disjoint GroupKFold evaluation. Class taxonomies and validation protocols differ across all entries, as shown in Table 6.

The entries in Table 6 reflect a wide range of evaluation strictness, from within-subject protocols that inflate performance by training and testing on the same individual, to cross-subject LOSO approaches that more closely approximate population generalisability. Published PAMAP2 and WESAD results consistently outperform the present framework on the same datasets because those methods apply dataset-native features extracted under task-specific protocols, whereas the RCG schema enforces a compact 13-feature deployment-oriented representation across all five repositories simultaneously. The present framework, therefore, does not claim competitive accuracy against per-dataset specialised pipelines; it claims coherence, portability, and deployment feasibility across heterogeneous repositories under a single fixed schema—a different and complementary evaluation target.

Among the evaluated unimodal settings, EMGGestures produced the strongest overall result, with Random Forest achieving 0.829 ± 0.060 accuracy and 0.785 ± 0.081 macro-F1, followed closely by SVM with an RBF kernel. PAMAP2 also yielded comparatively strong unimodal performance, particularly for the hand-mounted IMU stream, where Random Forest achieved 0.709 ± 0.036 accuracy and 0.649 ± 0.048 macro-F1. Within WESAD, the most informative unimodal stress signal was chest respiration, for which Random Forest reached 0.762 ± 0.047 accuracy and 0.557 ± 0.084 macro-F1. These results suggest that the present feature design is particularly effective when the target behaviour is strongly expressed through structured inertial dynamics or direct neuromuscular activation.

By contrast, the EEG-only repositories exhibited markedly weaker performance. In EEGMMIDB, SVM with an RBF kernel produced the strongest unimodal result, with 0.553 ± 0.007 accuracy and 0.531 ± 0.035 macro-F1. In DEAP, the best unimodal result was obtained with LDA, yielding 0.521 ± 0.009 accuracy and 0.489 ± 0.086 macro-F1. This weaker EEG behaviour is consistent with a structural limitation already anticipated in the analytical design: the deployment-consistent representation used here preserves compact spectral summaries, but not the richer spatial information on which many EEG decoding tasks more strongly depend. The lower EEG results should therefore be interpreted as a representation-task mismatch under deployment constraints rather than as evidence that the underlying datasets are intrinsically uninformative. Table 7 provides a dataset-level summary of the strongest unimodal result per repository, identifying the best-performing modality–classifier combination under subject-disjoint GroupKFold evaluation. Across the five repositories, sEMG- and IMU-dominant tasks produced the highest macro-F1 values, while EEG-only tasks yielded substantially lower scores under the fixed channel-averaged schema—a pattern discussed in detail in the full per-modality breakdown that follows.

**Figure 4:**
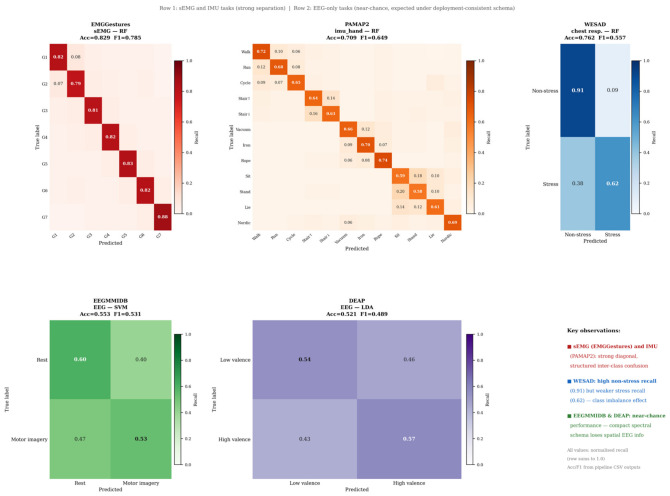
Representative confusion summaries for the strongest unimodal configurations across the evaluated public repositories. The panels illustrate class-wise prediction structure under subject-disjoint evaluation and highlight the contrast between higher-separation sEMG/IMU tasks and lower-separation EEG-only tasks under the deployment-consistent feature schema.

For each dataset, the entry reflects the modality–classifier combination achieving the highest macro-F1 across all configurations evaluated. Full per-modality results across all classifier families are reported in Table 5. The performance gap between the two task categories is consistent across classifier families and does not resolve with model substitution. In EMGGestures and PAMAP2, all three classifiers produce competitive results because the sEMG and IMU feature blocks preserve the discriminative structure relevant to the task. In EEGMMIDB and DEAP, no classifier recovers the spatial and temporal information that the compact spectral representation foregoes. This pattern establishes that the primary source of variation across datasets is representational rather than model-specific, which makes the unimodal tier a stable reference baseline for interpreting the multimodal and adaptation results that follow. The class-wise error structure of the strongest unimodal configurations is summarised in Figure 4.

Figure 4 reinforces the pattern already visible in Table 5. sEMG- and IMU-centred tasks show comparatively concentrated class structure, whereas EEG-only tasks exhibit broader confusion patterns consistent with weaker separability under the fixed representation. In this sense, the confusion analysis supports the broader interpretation that unimodal performance in the present pipeline is governed less by nominal modality label than by the degree to which the chosen feature schema preserves task-relevant discriminative structure.

Within the overall architecture, these unimodal results serve as the baseline reference against which later multimodal and adaptation-stage gains must be judged. They define the performance achievable before cross-modal complementarity, cross-domain adaptation, or deployment-specific model selection is introduced, and therefore provide the correct starting point for interpreting the multimodal fusion behaviour examined in the next subsection.

### 5.4. Multimodal Classification and ROC Analysis

Multimodal evaluation was performed only for repositories with multiple natively available physiological channels, namely WESAD and PAMAP2. In both cases, fusion was implemented at the feature level by concatenating modality-specific descriptors extracted within a shared analysis window, thereby preserving the deployment-consistent schema logic established earlier. The purpose of this stage was not simply to seek higher aggregate scores, but to determine whether complementary modalities improved class-balanced performance and decision stability relative to the strongest unimodal baselines under identical subject-disjoint evaluation conditions. The multimodal fusion results are summarised in Table 8.

Table 8 shows that multimodal fusion consistently improved macro-F1 over the strongest unimodal baseline in both datasets, although the magnitude and shape of that improvement depended on the task and classifier family. In WESAD, the largest class-balanced gain was observed for LDA under the full three-modality configuration, where macro-F1 increased from 0.356 to 0.497, yielding a gain of 0.141. SVM and Random Forest also improved under fusion, reaching 0.639 and 0.646 macro-F1, respectively. In PAMAP2, fusion of the two IMU streams already produced substantial improvement, and the addition of heart rate further increased performance, with the strongest result obtained by Random Forest at 0.830 accuracy and 0.740 macro-F1. These patterns indicate that multimodal fusion contributes more than a marginal averaging effect; it improves class-balanced discrimination under subject-disjoint evaluation when the contributing modalities capture genuinely complementary structure.

**Figure 5:**
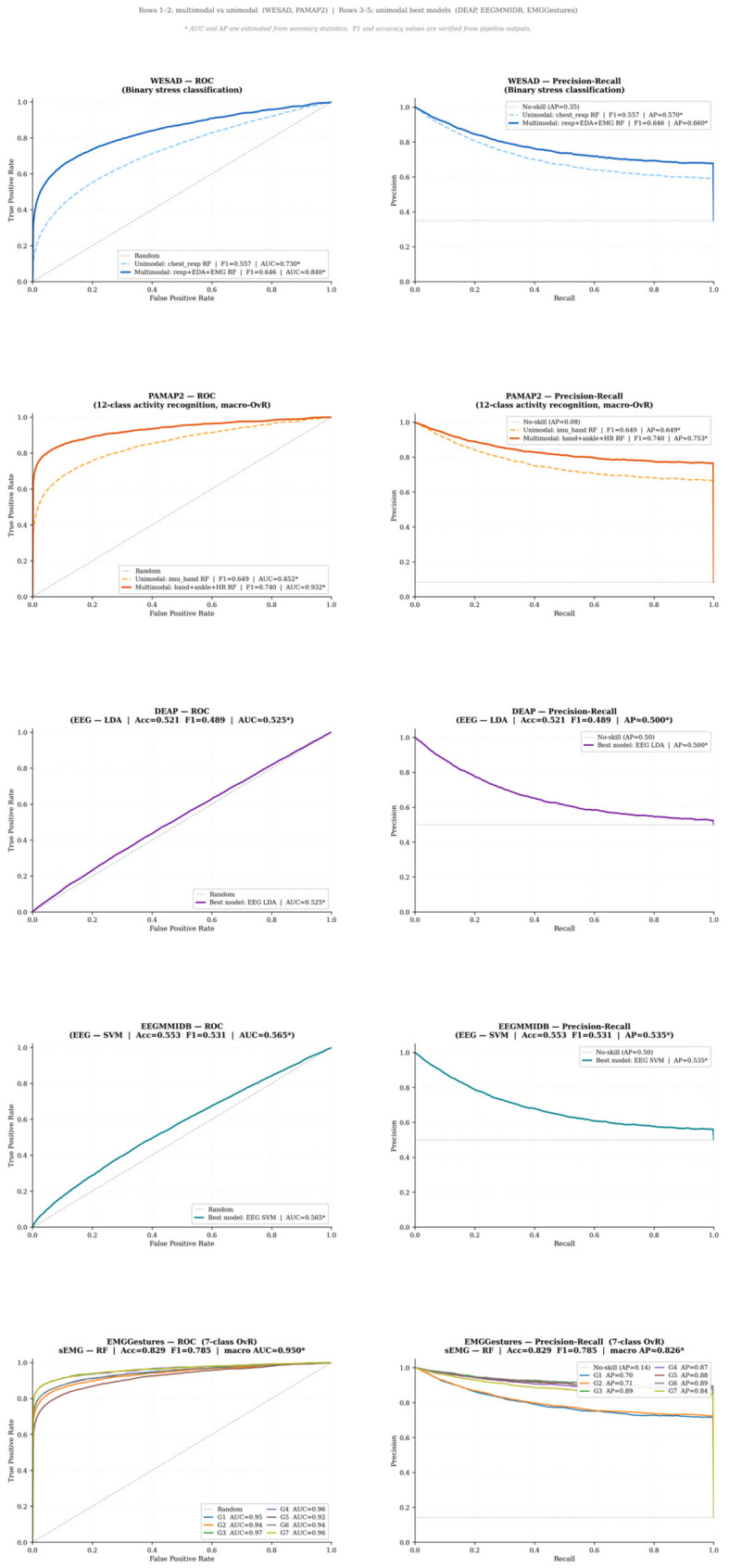
ROC and precision–recall summaries for multimodal WESAD and PAMAP2 configurations, together with the strongest unimodal models for DEAP, EEGMMIDB, and EMGGestures, under subject-disjoint evaluation. The panels illustrate threshold behaviour for the fused settings and highlight the improvement in decision separation relative to the weaker unimodal baselines.

The behaviour of the two datasets nevertheless differs in an instructive way. In WESAD, the gain arises from combining partially redundant but still complementary physiological channels in a comparatively low-dimensional stress-detection task. In PAMAP2, the gain is larger in absolute accuracy terms because the task is more structurally tied to coordinated motion context, and the paired IMU streams capture distinct but related views of the same activity state. Heart rate contributes a further stabilising signal, but the principal boost comes from cross-location inertial complementarity. This contrast supports the broader interpretation that multimodal benefit is governed not by modality count alone, but also by the degree of physiological and contextual complementarity among the fused channels. Receiver-operating and precision–recall behaviour for the multimodal WESAD and PAMAP2 settings, together with the strongest unimodal reference models for the remaining repositories, is summarised in Figure 5.

**Figure 6:**
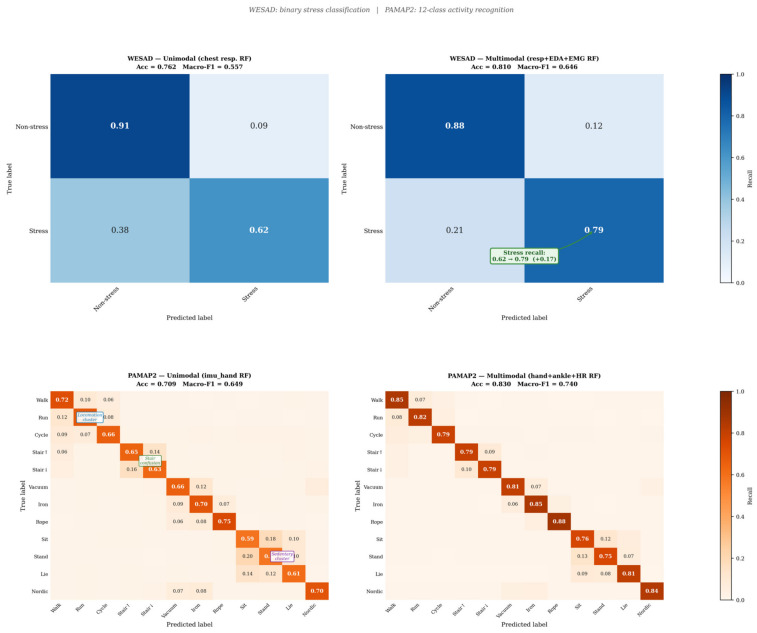
Confusion summaries for unimodal versus multimodal configurations in WESAD and PAMAP2 under subject-disjoint evaluation. The panels illustrate how fusion changes class-wise error structure relative to the unimodal baselines.

Figure 5 shows that the benefit of multimodal fusion is not confined to a single operating point. The strongest fused configurations exhibit broader class separation and more stable threshold behaviour than the weaker unimodal baselines, particularly in WESAD, where the fused models reduce the ambiguity associated with single-channel stress inference. In PAMAP2, the ROC and precision–recall profiles are correspondingly stronger, consistent with the higher absolute separability already seen in Table 6. These threshold-level views therefore support the same conclusion as the aggregate metrics: multimodal integration improves not only nominal score values but also the stability of the underlying decision surface. Confusion behaviour for the WESAD and PAMAP2 unimodal-to-multimodal comparison pairs is further summarised in Figure 6.

Taken together, the multimodal results confirm that feature-level fusion is compatible with the deployment-aware analytical logic of the pipeline while still yielding measurable gains over unimodal reference models. These gains are not uniform across tasks, but they are directionally consistent in both evaluated multimodal repositories. This makes multimodal fusion the first clear value-adding stage beyond the unimodal baseline layer and provides the immediate empirical basis for the cross-dataset and robustness interpretation developed in the next subsection.

### 5.5. Cross-Dataset and Robustness Analysis

The preceding results reveal a consistent cross-dataset pattern: robustness in the present pipeline depends less on nominal modality label than on the degree of structural alignment between the target task and the fixed deployment-consistent representation. Tasks dominated by amplitude-stable neuromuscular or inertial structure, such as EMGGestures and PAMAP2, remain comparatively robust under subject-disjoint evaluation, whereas EEG-only tasks remain markedly weaker under the compact channel-averaged schema used throughout the present study. WESAD occupies an intermediate position, with multimodal physiological fusion improving stability over single-channel stress inference without reaching the separability observed in the sEMG- and IMU-centred benchmarks. This pattern indicates that the constrained representation is most effective when the class structure is expressed through compact temporal or spectral summaries rather than through richer spatial structure that is not preserved in the present edge-compatible schema.

The same robustness pattern is visible when multimodal gains are examined relative to the classifier-matched unimodal baselines. In WESAD, the strongest fusion improvements were +0.141 for LDA, +0.122 for SVM, and +0.090 for RF under the full three-modality configuration. In PAMAP2, the corresponding gains were +0.109 for LDA, +0.079 for SVM, and +0.090 for RF when heart rate was added to the two-IMU configuration. These gains are directionally consistent across model families and across both multimodal repositories, indicating that the benefit of fusion is not tied to a single classifier or to an isolated dataset-specific effect. Rather, multimodal integration improves robustness when the contributing channels encode genuinely complementary physiological or contextual structure under the same fixed evaluation rules.

A second robustness question concerns whether modality-matched public covariance information can regularise the hardware-aligned LDA branch without breaking the schema and deployment contract of the pipeline. This question is not interpreted as a search for universal transfer superiority or semantic reuse across heterogeneous tasks. Instead, it is evaluated as a staged feasibility problem: whether public-domain covariance priors can provide a schema-compatible regularisation term, whether hardware-only fitting can recover stable discrimination under the full 17-feature contract, and whether the resulting artefacts remain deployable within the target resource envelope. Stage 1, therefore, produces modality-matched covariance priors and public-domain reference statistics, Stage 2 evaluates hardware adaptation under the shared and full schemas, and Stage 3 generates the edge and cloud deployment artefacts. The stage-wise outcomes are summarised in Table 9.

Table 9 shows that the staged procedure is best interpreted as a feasibility and schema-validation result rather than as evidence that public pretraining consistently improves hardware accuracy. At the 13-feature intersection, transfer LDA (F1 = 0.954, λ = 0.20) exceeded the hardware scratch baseline (F1 = 0.952, delta = +0.002), with lambda selected by GroupKFold CV on real session groups. The RF transfer branch falls back to the scratch configuration in both feature settings due to the class-space incompatibility between the pooled public label space and the 3-class hardware task (delta = 0.000, documented in Section 4.3). At the 17-feature level, transfer LDA (F1 = 0.948, λ = 0.40) again exceeded scratch LDA (F1 = 0.944, delta = +0.004), confirming that the modality-matched covariance prior contributes a small but consistently positive regularisation effect. The observed improvement, while modest in absolute magnitude, was directionally consistent across all five GroupKFold folds and is properly interpreted as a schema-compatibility feasibility demonstration rather than a performance claim. In this procedure, public repositories contribute only modality-matched covariance structure within the shared feature blocks; hardware class means, class priors, scaling parameters, and all label assignments are estimated exclusively from the hardware sessions, and no class-semantic information is transferred across the domain boundary. The pipeline does not converge to a single winner across all stages. Instead, different models dominate different operational roles: scratch SVM achieves the highest raw hardware-stage score (F1 = 0.952 at 17-feat), LDA is the preferred embedded artefact (716 B RAM), and RF remains the cloud-side model retained for longitudinal analytics and periodic retraining. The marginal LDA gain confirms that the public prior adds a small but positive representational contribution once the hardware-exclusive features re-anchor the adapted model to the deployment distribution. For the hardware evaluation branch, all reported performance metrics (Table 9, Stage II–III rows) were derived using session-disjoint GroupKFold cross-validation (k = 5, leave-one-session-out) across five controlled bench sessions. Window size is 5.0 s (1250 samples at 250 Hz) with a stride of 2.0 s. Each fold holds out one complete session as the test partition while training on the remaining four, preventing any leakage across the session boundary. The hardware F1 values reported in Table 9, therefore, reflect cross-session generalisation within the controlled bench acquisition regime.

**Figure 7:**
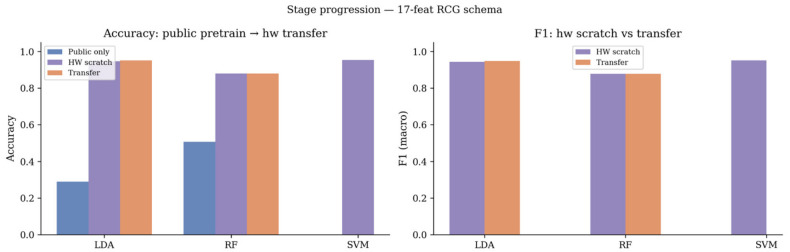
Stage progression of the RCG adaptation pipeline under the final 17-feature schema. The left panel compares public covariance-estimation outputs, hardware-scratch accuracy, and covariance-regularised hardware accuracy for the evaluated classifier branches, whereas the right panel contrasts hardware-scratch and regularised macro-F1 values. The figure shows that modality-matched public covariance information provides only a limited regularisation prior, while final deployment-facing behaviour remains determined by hardware-aligned learning under session-disjoint evaluation across five bench sessions.

This behaviour clarifies the limited role of the public branch within the present workflow. The public repositories provide modality-matched covariance information and broader feature-distribution context, but they do not supply class semantics or target-task decision structure for the hardware problem. The hardware sessions determine all deployment-facing discrimination under the full 17-feature schema because class means, priors, scaling parameters, labels, and hardware-exclusive variables are estimated from the bench-acquired data. The resulting stage progression, expressed as public covariance estimation, hardware-scratch fitting, and covariance-regularised hardware fitting, is summarised in Figure 7.

Figure 7, therefore, distinguishes the two evidential roles within the adaptation pathway. The public branch contributes modality-matched covariance priors and reference feature distributions, whereas the bench-acquired hardware sessions determine whether those priors provide any measurable regularisation benefit under the full 17-feature contract. The observed LDA gain is small and does not establish generic transfer superiority, while the RF branch produces a null transfer result under class-space incompatibility. The strongest supported claim is therefore limited to schema-compatible covariance regularisation and hardware-anchored deployment feasibility.

### 5.6. Explainability and Attribution Results

Explainability analysis was performed to determine whether the performance patterns reported in the preceding subsections were supported by physiologically plausible feature use rather than by opaque dataset-specific fitting. To keep interpretability distinct from predictive performance, attribution was analysed separately for the multimodal public-data branch and for the hardware-aligned full-schema branch. In the public branch, SHAP-based attribution was computed for the strongest fused Random Forest configurations in WESAD and PAMAP2. In the hardware branch, feature importance was examined for the final 17-feature deployment-oriented model using the ranking produced during the Stage IIb analysis. Together, these views provide complementary evidence regarding which modalities and features drive classification behaviour under the pipeline.

For WESAD, attribution concentrated primarily in the respiration and EMG groups, with EDA contributing a smaller but still non-negligible share. Summed mean absolute SHAP values were highest for respiration-derived features, followed closely by EMG, whereas EDA ranked third. At the individual-feature level, the strongest contributors were chest_resp_f6 (0.115), chest_emg_f6 (0.079), chest_emg_f4 (0.050), chest_resp_f3 (0.045), and chest_eda_f1 (0.042). This distribution is consistent with the unimodal and fusion results reported earlier: respiration carries the most stable stress-related information under the present feature schema, while EMG and EDA provide complementary support that improves class-balanced discrimination under fusion.

**Figure 8:**
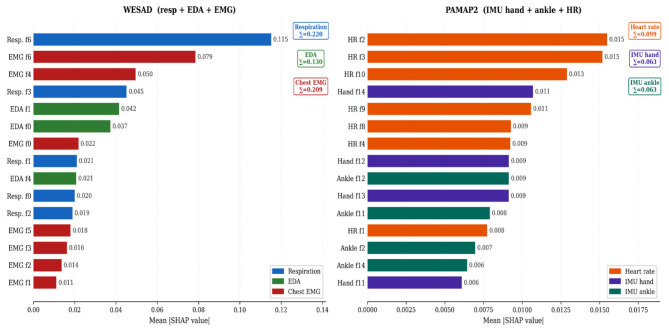
SHAP-based modality and feature attribution summaries for the strongest fused Random Forest configurations in WESAD and PAMAP2. The panels show mean absolute contribution rankings and highlight the concentration of importance within task-aligned modality groups.

For PAMAP2, attribution was distributed across all three fused streams, but the largest aggregate contribution arose from the heart-rate channel, with the two IMU branches contributing at comparable secondary levels. The strongest individual features were heart_rate_f2 (0.015), heart_rate_f3 (0.015), heart_rate_f10 (0.013), imu_hand_f14 (0.011), and heart_rate_f9 (0.011). The attribution structure, therefore, indicates that the multimodal gain in PAMAP2 is not driven solely by inertial redundancy. Rather, the heart-rate channel contributes a stabilising physiological context, while the two spatially separated IMU streams provide the dominant movement representation. This pattern aligns with the fusion results in Section 5.4, where the addition of heart rate improved the already strong two-IMU configuration. The principal public-branch attribution patterns are summarised in Figure 8.

Figure 8 reinforces that multimodal gains in the present pipeline are physiologically structured rather than uniformly distributed across all fused inputs. In WESAD, attribution concentrates in respiration and EMG, which matches the stronger role of these channels in stress discrimination. In PAMAP2, attribution is shared across motion and heart-rate features, consistent with the interpretation that activity recognition benefits from both kinematic and physiological context. These results therefore support the claim that feature-level fusion in the pipeline retains interpretable modality structure rather than collapsing into an opaque aggregate predictor.

**Figure 9:**
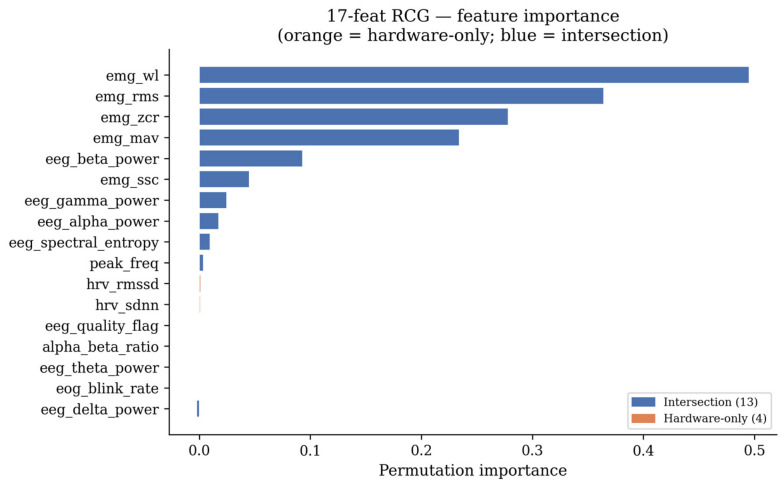
Feature importance for the final 17-feature RCG hardware branch, shown as permutation importance and grouped by intersection features versus hardware-only variables. The dominant contributors are emg_wl, emg_rms, emg_zcr, and emg_mav, followed by eeg_beta_power. In contrast, the hardware-exclusive variables related to HRV, EOG blink rate, and EEG quality remain negligible under the current bench-acquired data regime.

For the full 17-feature hardware-aligned branch, feature importance was re-estimated on the current pipeline outputs using the final hardware schema rather than the older SHAP-based transfer summary. The updated ranking shows a strongly concentrated importance profile dominated by the EMG time-domain descriptors, led by emg_wl, emg_rms, emg_zcr, and emg_mav, with eeg_beta_power emerging as the principal EEG contributor. The remaining spectral EEG variables contribute at lower but non-zero levels, whereas the hardware-exclusive variables derived from HRV, EOG, and the EEG quality flag remain near-zero under the present five-session bench regime. This pattern indicates that the full-schema hardware branch is being driven primarily by stable EMG discriminants, with a secondary contribution from compact EEG spectral structure, rather than by broad, uniformly distributed attribution across all 17 variables. The resulting hardware-aligned ranking is summarised in Figure 9.

Taken together, the attribution results show that the pipeline remains interpretable across both the public multimodal branch and the hardware-aligned branch, but not in the same way. In the public multimodal setting, the dominant features reflect task-aligned complementarities across modality groups. In the hardware branch, however, the final 17-feature ranking is much more sharply concentrated, with EMG carrying most of the discriminative burden and EEG contributing a smaller spectral support layer. The hardware-exclusive variables do not vanish from the schema, but under the present bench conditions, they do not yet emerge as major drivers of classification. This strengthens the results narrative by showing that the observed performance profile is structurally coherent with the sensing design: the strongest signal comes from the modalities expected to dominate under controlled bench acquisition, while the auxiliary contextual variables remain available for richer future regimes without needing to alter the deployment contract.

### 5.7. Summary of Experimental Findings

Taken together, the experimental results support four main findings. First, unimodal performance was strongest in tasks whose class structure was well aligned with compact, deployment-consistent feature summaries, particularly sEMG gesture recognition and IMU-based activity classification. Second, multimodal feature-level fusion produced consistent class-balanced gains in the two repositories that exposed genuinely complementary modality combinations, namely WESAD and PAMAP2. Third, the staged public-to-hardware training pathway was valuable primarily as a schema-consistent feasibility route rather than as evidence that heterogeneous public pretraining universally improves hardware accuracy. Fourth, the final deployment artefacts confirmed that high classification performance could be retained under a resource-constrained embedded target without breaking continuity with the broader cloud-side analytical workflow. These themes are consistent with the experimental progression already defined for Section 5 in the manuscript structure.

At the public-benchmark level, the strongest unimodal baseline arose in EMGGestures, where Random Forest reached approximately 0.829 accuracy and 0.785 macro-F1, whereas EEG-only tasks remained near chance or only modestly above it under the channel-averaged deployment representation. In the multimodal branch, WESAD showed a best fusion gain of 0.1407 macro-F1 over the strongest unimodal baseline, and PAMAP2 showed a best gain of 0.109. These improvements were directionally consistent across classifier families and therefore support the claim that multimodal benefit in the present pipeline reflects physiologically meaningful complementarity rather than an isolated model-specific effect. The deployment-facing comparison is summarised in Table 10.

Table 10 shows that the preferred edge artefact is not simply the numerically strongest model, but the strongest model that remains compatible with the deployment envelope. Edge LDA Pareto-dominates the quantised edge Random Forest, achieving higher macro-F1 (0.9435 vs. 0.8184) while requiring 10.4× less RAM (716 B vs. approximately 7440 B), reinforcing that the linear model is not merely the deployable choice but the analytically superior one on both dimensions simultaneously under the present bench evaluation. By contrast, the cloud-resident Random Forest produced the highest overall performance under the full 17-feature schema and therefore remains the appropriate target for longitudinal aggregation and periodic retraining. This division is fully consistent with the asymmetric edge–cloud strategy formalised earlier, in which lightweight bounded inference is prioritised on-device while broader analytical capacity is retained upstream.

These deployment results reflect cross-session generalisation within a controlled bench acquisition regime. The hardware comparisons are based on session-disjoint GroupKFold evaluation across 1490 windows from five sessions with heterogeneous signal characteristics, providing a more robust estimate of hardware branch performance than any single-session protocol. The hardware scores should be read as evidence that the analytical contract remains executable, schema-consistent, and generalisable across session-level variability in the bench context, not as evidence of population-scale wearable validation. With that boundary kept explicit, the experiments collectively support the central claim of this paper: the proposed analytics layer is reproducible, deployment-aware, and structurally capable of linking heterogeneous public benchmarks to embedded multimodal inference within a single coherent edge–cloud pipeline.

## 6. Discussion

The results demonstrate that the principal value of the proposed framework lies in maintaining analytical continuity across heterogeneous public datasets, multimodal fusion, hardware-aligned modelling, and embedded deployment. The study does not establish state-of-the-art performance on any individual benchmark. Instead, it shows that repository-native biosignal analyses, modality-matched public covariance priors, session-disjoint hardware evaluation, and schema-locked edge inference can be organised within one reproducible edge–cloud workflow.

### 6.1. Key Findings

The central finding of this study is that multimodal physiological analytics for portable biosensor systems can be made deployment-consistent without collapsing into either benchmark-driven optimisation or hardware-driven oversimplification. The results show that the pipeline remains analytically coherent across heterogeneous public repositories, multimodal fusion settings, hardware-aligned adaptation, and final edge–cloud deployment, even though these stages operate under different data regimes and computational assumptions. The larger significance of the findings, therefore, lies not in any single metric peak, but in demonstrating that a fixed-schema workflow can preserve continuity from public-domain modelling to embedded inference without breaking interpretability or operational validity.

Second, multimodal fusion improved class-balanced performance in WESAD and PAMAP2. The largest classifier-matched macro-F1 gains were 0.141 for WESAD and 0.109 for PAMAP2, while the gains obtained by comparing the best fused and best unimodal configurations overall were approximately 0.090 in both repositories. These results indicate that multimodality is useful when channels contribute complementary physiological or contextual information. Additional modalities do not provide value merely by increasing input dimensionality.

Third, modality-matched public covariance priors produced only modest regularisation gains in the hardware LDA branch. The macro-F1 improvement over hardware-only LDA was approximately 0.002 in the 13-feature intersection and 0.005 under the complete 17-feature schema. These changes were directionally consistent across grouped folds but are too small to support a general claim of transfer superiority. The supported interpretation is narrower: public covariance information can be introduced without transferring incompatible class semantics or breaking the hardware schema.

Finally, the deployment results support asymmetric model placement. The LDA artefact achieved strong cross-session performance with an estimated RAM footprint of approximately 716 B and measured inference latency of 111.1 ± 0.9 µs. The cloud RF did not exceed the edge LDA numerically in the present evaluation, but it remains useful for longitudinal analysis, model comparison, and periodic retraining. Edge and cloud models, therefore, need not be identical or ordered solely by predictive score; they may be selected according to distinct operational responsibilities while remaining connected through a common deployment contract.

### 6.2. Comparison with Prior Work

The present findings align with prior work in showing that non-invasive biosignal classification performance depends strongly on both modality structure and analytical design, but they also extend that literature in a more deployment-conscious direction. Much of the existing work on wearable and portable biosensor analytics has been reported within modality-specific or task-specific silos, with strong results often demonstrated either in narrowly defined unimodal settings or under computational assumptions that do not translate directly to embedded inference. By contrast, the framework developed here evaluates heterogeneous public repositories under a single subject-disjoint, schema-constrained, edge-aware workflow. The resulting comparison is therefore less about outperforming individual benchmark studies on their own terms than about showing that multimodal physiological analytics can remain coherent across repositories, tasks, and deployment tiers without being reformulated separately for each case.

Relative to conventional unimodal biosignal pipelines, the present results support a more qualified interpretation of performance. Prior work has repeatedly shown that sEMG- and IMU-based tasks are often more readily separable than EEG-only tasks under compact feature-based models, whereas EEG decoding frequently benefits from richer spatial information or more flexible representation learning [66]. The present results are consistent with that broader pattern. However, rather than treating the weaker EEG outcomes as a failure to match more expansive offline approaches, this study makes explicit that such behaviour follows from the deployment contract itself. The fixed feature schema and bounded edge-facing inference pathway intentionally privilege compact, stable descriptors over higher-dimensional or more spatially expressive representations [67]. In this sense, the work differs from benchmark-maximising studies by treating representational compression not as a downstream optimisation step, but as an upstream design principle that shapes the entire analytical stack.

The multimodal results also position the present study somewhat differently from prior fusion-focused literature. Many earlier multimodal biosignal studies have shown that combining channels can improve robustness [37], particularly in stress recognition, affective-state analysis, or activity monitoring. The current findings agree with that general conclusion, but they refine it by showing that multimodal benefit is not a universal consequence of adding modalities. Instead, the magnitude and direction of gain depend on whether the fused channels provide genuinely complementary physiological or contextual structure under the same constrained feature space. The results from WESAD and PAMAP2 therefore support a more selective interpretation of multimodality: fusion is most useful when it stabilises inference under heterogeneous sensing conditions, not when it merely increases channel count.

A further point of comparison concerns model complexity. A considerable part of the recent biosignal-learning literature has shifted toward deep architectures capable of learning directly from minimally processed signals, often with strong offline performance. The present work does not compete with that strand on raw representational expressiveness. Instead, it demonstrates that lightweight classifiers operating on interpretable, low-overhead feature sets can still produce analytically meaningful performance when the goal is not unrestricted benchmark optimisation but bounded inference under portable deployment constraints. This distinction is important [68,69] because it clarifies that the contribution of the pipeline is not to replace high-capacity offline models in every setting, but to define a reproducible pathway for cases where determinism, memory footprint, and update control are part of the problem rather than external engineering details.

The clearest divergence from much prior work lies in the treatment of edge–cloud interaction. Hybrid architectures are widely discussed in health IoT and portable sensing research, but they are often described at a conceptual or systems level without explicitly showing how schema continuity, adaptation logic, and deployment validation are maintained across the edge–cloud boundary. In the present study, that boundary is treated as analytically constitutive. Public-domain modelling, multimodal fusion, hardware-aligned refinement, and final artefact export are linked through a common representational contract rather than through ad hoc transitions between offline experimentation and deployment. The resulting contribution is therefore less a claim of isolated state-of-the-art accuracy than a claim of methodological continuity: compared with prior work, the framework more explicitly binds model development, multimodal evaluation, and deployment realisation into a single structured analytical pathway.

### 6.3. Limitations and Challenges

The first limitation is representational capacity. The fixed 17-feature schema supports bounded inference and reproducible deployment but cannot preserve every property of the source signals. This is most apparent in the EEG branch. The current hardware acquires one EEG channel, so multichannel covariance, inter-electrode asymmetry, connectivity, spatial filtering, and source-level descriptors cannot be calculated. The schema does not discard spatial information generated by the device; the evaluated acquisition configuration does not produce it. Extending the framework to spatial EEG analysis would therefore require a multichannel front end, defined electrode topology, revised features, retraining, and renewed deployment validation. A second limitation arises from the heterogeneity of the public repositories themselves. User-independent stress detection remains materially harder than participant-specific modelling, and between-subject physiological variability can dominate reported gains if evaluation design is not carefully controlled. This is one reason the present work prioritises grouped, anti-leakage validation over record-wise splits [70]. Although the five selected datasets provide broad coverage across modality classes and application domains, they remain fundamentally non-uniform in acquisition protocol, label semantics, subject population, and recording context. As a result, cross-dataset comparisons in the present study should be interpreted as structured analytical contrasts rather than as strictly commensurate performance contests. The shared 13-feature intersection permits a common processing pathway, but it does not erase the deeper differences between repositories [71]. This limitation is especially relevant for the staged adaptation branch, where public-domain breadth is useful for defining a common representational envelope, but does not by itself guarantee semantically clean transfer into the hardware-facing task space.

A third challenge concerns the scope of the hardware evaluation regime. The hardware branch of the present study does not incorporate multi-subject recruitment. The reported session-disjoint GroupKFold results are bound to five bench-acquired sessions conducted by a single operator under controlled laboratory conditions. No on-body, ambulatory, or multi-subject validation has been performed, and generalisation of the analytical findings to broader populations cannot be inferred without dedicated multi-subject wearable validation studies. The hardware-aligned branch is evaluated across five bench sessions comprising 1490 windows under session-disjoint GroupKFold cross-validation, which establishes cross-session robustness within the controlled bench acquisition context. The distinction between public-dataset evidence and hardware-branch evidence, therefore, remains essential: the former speaks to subject-disjoint behaviour across established physiological repositories; the latter speaks to whether the analytical contract remains stable across session-level signal variability in a bench deployment context. Progression to real hardware acquisition under ethics-approved and regulatorily appropriate protocols remains the necessary next step for establishing wearable-use generalisability.

The relative advantage of LDA over tree-based and kernel methods in the present study should additionally be read as regime-specific. The current bench acquisition context favours a compact linear decision boundary; with a larger session inventory, richer within-session non-linear structure, and more stable cross-session recurrence of contextual features such as HRV and EOG, RF and SVM could be expected to become more competitive or to surpass the linear baseline. Motion-artefact tolerance has not been empirically characterised in this study. The bench sessions were acquired under controlled static conditions, and the behaviour of the LDA edge artefact under artefact-corrupted inputs arising from physical movement, electrode displacement, or cable motion has not been evaluated. Artefact-robust front-end design and feature-level signal validation are identified as prerequisites for future ambulatory validation.

A further limitation lies in the current adaptation strategy itself. The staged public-to-hardware pathway succeeds as a schema-consistent bridge, but it does not resolve the deeper problem of heterogeneous label spaces across pooled public datasets. The public branch spans stress, affect, activity, gesture, and motor-imagery tasks that are analytically useful when treated as heterogeneous pretraining sources, yet they do not form a unified semantic label hierarchy that can be transferred cleanly into a compact hardware task definition [72]. This is why the strongest claim supported by the adaptation results is one of pipeline continuity and feasibility rather than universal transfer advantage. The present study, therefore, demonstrates that a deployment-consistent bridge can be built, but not that the current pooled-label formulation is the final or optimal solution for cross-domain biosignal transfer [73].

Finally, the broader challenge is one of scope. The present work deliberately isolates the analytics layer from broader questions of long-duration wearability, user variability under unconstrained movement, clinical interpretation, and longitudinal real-world monitoring. That narrowing was necessary to keep the contribution methodologically precise, but it also means that the current manuscript stops short of the settings in which many portable biosensor systems would ultimately need to operate. The framework established here should therefore be understood as an analytical foundation rather than as a fully closed translational solution. Its value lies in making the constraints visible, formalising a reproducible edge–cloud workflow, and defining a disciplined basis on which more realistic multi-subject and wearable-facing validation can later be built.

### 6.4. Future Directions

The most immediate next step is to extend the present framework from cross-session bench validation to multi-subject hardware validation under a deployment-relevant physiological recording regime. The current hardware branch already demonstrates that the fixed 17-feature schema, staged adaptation logic, telemetry-supported contextual analytics, and asymmetric edge–cloud workflow remain coherent across five session-disjoint bench acquisitions. What it does not yet establish is how this analytical contract behaves across subjects, electrode variability, motion conditions, and longer-duration on-body recordings. Such a study would also be the appropriate context for characterising system-level power consumption and duty-cycled acquisition strategies, which require all hardware components to be integrated into a single powered assembly and cannot be meaningfully addressed in the present bench configuration.

A second future direction concerns the representational limitations of the current schema, particularly for EEG-dominant tasks. The weaker cortical results observed here suggest that compact channel-averaged spectral summaries are not sufficient to preserve all of the structure required for more demanding EEG inference. Future work should therefore examine whether richer but still deployment-compatible extensions of the schema can recover more spatially informative cortical features without breaking the bounded execution envelope of the edge tier [74,75]. This does not necessarily imply abandoning the fixed-schema philosophy; rather, it suggests that the present 17-feature design may represent one feasible operating point within a broader family of edge-aware biosignal representations.

A third direction concerns cross-domain training itself. The results of the staged public-to-hardware pathway indicate that the shared 13-feature intersection is effective as a continuity-preserving bridge, but not yet as a semantically principled transfer framework across heterogeneous physiological tasks. A more mature version of this pipeline would require either explicit label remapping into a common analytical hierarchy or a multi-task training design in which dataset-specific output spaces are preserved while the encoder or feature backbone is shared across repositories [64]. Such extensions would allow public-domain breadth to be used more meaningfully without forcing unlike tasks into an overly coarse pooled label structure. In that sense, the next generation of the framework should treat cross-domain learning not merely as statistical reuse, but as a representational design problem in its own right [76].

Another important extension lies in lifecycle intelligence. The present pipeline already separates edge inference from cloud-side retraining and version control, but future work could formalise this further through scheduled model monitoring, drift detection, schema-version management, and controlled rollback logic under changing acquisition conditions. This would move the framework from a deployment-aware analytical stack toward a more complete operational learning system, while still respecting the asymmetry that makes embedded inference practical. Such evolution is particularly relevant for portable and wearable biosensor settings, where sensor conditions, user behaviour, and recording environments are unlikely to remain stationary over time [77]. Parallel advances in soft and biohybrid neural interfaces indicate that future electrophysiological systems will increasingly prioritise conformability, long-term signal stability, and biological integration alongside analytical performance. Although the present platform remains non-invasive, these developments define a broader interface-design trajectory within which future RCG embodiments may evolve [78].

A successful next-stage programme should reconnect the present analytical layer to the wearable-facing and healthcare-accessibility trajectory that motivates the broader project. The first stage should comprise multi-subject on-body acquisition under ethics-approved protocols, repeated-session robustness testing, motion-artefact characterisation, electrode-placement variability assessment, longer-duration recordings, body-proximal and ambulatory deployment scenarios, and statistical power analysis sufficient to support population-level generalisation claims. This stage should preserve the schema contract, evaluation logic, and edge–cloud division of labour established in the present study while determining how the analytical pipeline behaves under realistic physiological and operational variability.

The second stage should address integrated system performance, including complete power profiling, duty-cycled acquisition, communication reliability, power autonomy, usability, maintainability, and resilience under intermittent connectivity. These factors cannot be inferred from classifier performance alone and require evaluation of the Nano, analogue front ends, storage, display, auxiliary sensors, and LTE gateway as a single operational assembly.

A subsequent translational stage should evaluate the system against accepted reference measurements within clearly defined health-monitoring use cases, followed by population-specific validation, clinical feasibility assessment, data-governance review, and regulatory pathway evaluation appropriate to the intended use. Cost-effective deployment should be treated as an explicit engineering objective. The modular architecture adopted here provides a practical basis for this progression by assigning lightweight inference to a microcontroller-class edge node, transmitting compact feature representations rather than continuous raw signals, and retaining computationally intensive training and longitudinal analysis in the cloud. Future accessibility studies should therefore quantify component cost, connectivity requirements, maintenance burden, cloud dependence, and usability across settings with different infrastructure constraints. If these stages are completed successfully, the present work will provide a stable analytical foundation for clinically relevant, accessible, and cost-conscious portable biosensor deployment.

## 7. Conclusions

This study presented the machine-learning and analytics layer of the RCG architecture as a deployment-aware framework for portable multimodal biosensor systems. Rather than treating model development, multimodal fusion, hardware adaptation, and embedded inference as separate problems, the proposed approach organised them within a single analytical workflow defined by a fixed feature schema, subject-disjoint public evaluation, and an explicitly asymmetric edge–cloud partition. In doing so, the work addressed a methodological gap that persists across much of the biosensor-learning literature, namely the lack of continuity between heterogeneous public benchmarking, multimodal physiological analysis, and deployment-facing model realisation under constrained hardware assumptions.

Across the evaluated public repositories, the results showed that the proposed pipeline remains most effective when the target task is well aligned with compact temporal and spectral descriptors, as observed in the sEMG and inertial benchmarks [79]. Multimodal feature-level fusion improved class-balanced performance in the repositories where complementary physiological channels were available, supporting the view that multimodality is most valuable when it stabilises inference under heterogeneous sensing conditions rather than when it simply increases input count [80]. The staged public-to-hardware pathway further demonstrated that heterogeneous benchmark modelling and hardware-facing refinement can be linked through a shared representational contract, even though the strongest claim supported by the present results is one of schema continuity, cross-session deployment stability, and deployment feasibility rather than universal transfer superiority. Finally, the deployment comparison confirmed that lightweight embedded inference and higher-capacity cloud analytics can remain analytically consistent without requiring identical model forms at both tiers.

Taken together, these findings support the main conclusion of the paper: portable multimodal biosensor analytics can be made reproducible, interpretable, and deployment-consistent when feature-schema stability, evaluation logic, and computational partitioning are treated as first-order design constraints rather than as downstream implementation details. The present work, therefore, contributes not a benchmark-maximising pipeline, but a structured analytical foundation for future multi-subject, wearable-facing biosensor systems in which embedded inference, cloud-supported adaptation, and multimodal physiological interpretation must coexist within the same operational framework.

## Figures and Tables

**Table 1:** Dataset-to-application alignment summary for the five public biosignal repositories used in the present study.

Dataset	Primary Modality/Modalities	Native Task Formulation	Motor Intent Recognition	Affective-State Monitoring	Physiological Stress Assessment	Activity Classification	Gesture-Based Interaction	Neurophysiological State Analysis
WESAD	Chest respiration, EDA, EMG	Binary stress vs. non-stress	Low	Moderate	High	Low	Low	Moderate
DEAP	EEG	Binary valence classification	Low	High	Low	Low	Low	Moderate
PAMAP2	IMU, heart rate	12-class activity recognition	Low	Low	Low	High	Low	Moderate
EMGGestures	sEMG	7-class gesture recognition	Moderate	Low	Low	Low	High	Moderate
EEGMMIDB	EEG	Binary motor imagery classification	High	Low	Low	Low	Low	High

**Table 2:** Dataset-specific processing and evaluation protocol used in the public benchmark branch of the analytics pipeline.

Dataset	Modality/Modalities Used	Native Task Formulation	Window Length	Stride/Overlap	Split Policy
WESAD	Chest respiration, EDA, EMG	Binary stress classification	5 s	2.5 s stride	Subject-disjoint GroupKFold
DEAP	EEG	Binary valence classification	4 s	2 s stride	Subject-disjoint GroupKFold
PAMAP2	IMU (hand, ankle), heart rate	12-class activity classification	5.12 s	1 s stride	Subject-disjoint GroupKFold
EMGGestures	sEMG	7-class gesture classification	250 ms	50% overlap	Subject-disjoint GroupKFold
EEGMMIDB	EEG	Binary motor imagery classification	2 s	1 s stride	Subject-disjoint GroupKFold

**Table 3:** Edge deployment profile of the LDA artefact on the Arduino Nano RP2040 Connect.

Metric	Value
Model RAM footprint	716 B
Measured inference latency (mean ± SD)	111.1 ± 0.9 µs
Estimated active current	28.0 mA
Estimated energy per inference	10.26 µJ
Average inference-compute contribution	2.05 µW

**Table 4:** Operational summary of the machine-learning stack.

Stage	Primary Function	Active Schema	Computational Tier	Principal Outputs
Section 4.1 Public baseline construction	Dataset loading, windowing, and unimodal baseline evaluation	13-feature intersection	Cloud	Baseline results and dataset-specific reference statistics
Section 4.2 Fusion and ablation analysis	Multimodal combination, ablation comparison, and attribution analysis	Dataset-native within shared workflow	Cloud	Fusion results, ablation deltas, and attribution outputs
Section 4.3 Joint training and cross-domain adaptation	Public pretraining and hardware-aligned adaptation	13-feature intersection → 17-feature full schema	Cloud	Adapted LDA and schema-consistent hardware-aligned models
Section 4.4 Deployment artefact generation	Model export, schema validation, deployment gating, and update logic	17-feature full schema	Edge + Cloud	Edge inference artefact and cloud-resident classifier

**Table 5:** Unimodal classification performance across the five public repositories under subject-disjoint evaluation.

Dataset	Modality	Model	Accuracy	Macro-F1
EMGGestures	sEMG	LDA	0.763 ± 0.063	0.658 ± 0.056
EMGGestures	sEMG	SVM (RBF)	0.815 ± 0.066	0.772 ± 0.087
EMGGestures	sEMG	RF	0.829 ± 0.060	0.785 ± 0.081
WESAD	Chest respiration	LDA	0.737 ± 0.040	0.356 ± 0.161
WESAD	Chest respiration	SVM (RBF)	0.763 ± 0.050	0.517 ± 0.106
WESAD	Chest respiration	RF	0.762 ± 0.047	0.557 ± 0.084
WESAD	Chest EDA	LDA	0.765 ± 0.053	0.343 ± 0.249
WESAD	Chest EDA	SVM (RBF)	0.718 ± 0.075	0.431 ± 0.123
WESAD	Chest EDA	RF	0.641 ± 0.115	0.447 ± 0.087
WESAD	Chest EMG	LDA	0.672 ± 0.044	0.169 ± 0.059
WESAD	Chest EMG	SVM (RBF)	0.667 ± 0.066	0.351 ± 0.144
WESAD	Chest EMG	RF	0.649 ± 0.053	0.345 ± 0.113
PAMAP2	IMU hand	LDA	0.627 ± 0.022	0.565 ± 0.027
PAMAP2	IMU hand	SVM (RBF)	0.700 ± 0.037	0.634 ± 0.054
PAMAP2	IMU hand	RF	0.709 ± 0.036	0.649 ± 0.048
PAMAP2	IMU ankle	LDA	0.572 ± 0.023	0.528 ± 0.020
PAMAP2	IMU ankle	SVM (RBF)	0.613 ± 0.017	0.574 ± 0.017
PAMAP2	IMU ankle	RF	0.644 ± 0.013	0.634 ± 0.018
PAMAP2	Heart rate	LDA	0.335 ± 0.074	0.274 ± 0.054
PAMAP2	Heart rate	SVM (RBF)	0.360 ± 0.078	0.298 ± 0.048
PAMAP2	Heart rate	RF	0.344 ± 0.063	0.301 ± 0.055
EEGMMIDB	EEG	LDA	0.551 ± 0.008	0.522 ± 0.042
EEGMMIDB	EEG	SVM (RBF)	0.553 ± 0.007	0.531 ± 0.035
EEGMMIDB	EEG	RF	0.549 ± 0.011	0.510 ± 0.049
DEAP	EEG	LDA	0.521 ± 0.009	0.489 ± 0.086
DEAP	EEG	SVM (RBF)	0.515 ± 0.004	0.454 ± 0.095
DEAP	EEG	RF	0.511 ± 0.004	0.466 ± 0.077

**Table 6:** Representative literature benchmarks for the public datasets used in this study. Reported methods, evaluation protocols, class formulations, cohort sizes, and accuracies are reproduced from the cited sources.

Dataset	Reference	Method	Eval Protocol	Classes	Accuracy
WESAD	[21]	RF	LOSO, N = 15	3	0.930
WESAD	[63]	DNN	LOSO, N = 15	3	0.952
PAMAP2	[23]	Decision tree	LOSO, N = 9	12	0.841
EMGGestures	[24]	SVM	Within-subject	7	~0.900
EEGMMIDB	[64]	Multi-branch CNN	Cross-subject, N = 109	2	0.878
EEGMMIDB	[65]	Subject-indep. SSDA	Cross-subject, N = 105	2	~0.750

**Table 7:** Best unimodal classification result per public repository under subject-disjoint GroupKFold evaluation (k = 5).

Dataset	Best Modality	Best Model	Accuracy	Macro-F1
EMGGestures	sEMG	RF	0.829	0.785
WESAD	Respiration	RF	0.762	0.557
PAMAP2	IMU hand	RF	0.709	0.649
EEGMMIDB	EEG	SVM	0.553	0.531
DEAP	EEG	LDA	0.521	0.489

**Table 8:** Multimodal fusion performance and improvement over the best unimodal baseline under subject-disjoint evaluation.

Dataset	Modality Set	Model	Accuracy	Macro-F1	Classifier-Matched Unimodal Macro-F1	Fusion Delta (Macro-F1)
WESAD	chest_resp + chest_eda	LDA	0.779 ± 0.057	0.469 ± 0.216	0.356	+0.112
WESAD	chest_resp + chest_eda	SVM (RBF)	0.805 ± 0.022	0.636 ± 0.089	0.517	+0.119
WESAD	chest_resp + chest_eda	RF	0.791 ± 0.029	0.622 ± 0.076	0.557	+0.065
WESAD	chest_resp + chest_eda + chest_emg	LDA	0.763 ± 0.037	0.497 ± 0.159	0.356	+0.141
WESAD	chest_resp + chest_eda + chest_emg	SVM (RBF)	0.798 ± 0.034	0.639 ± 0.098	0.517	+0.122
WESAD	chest_resp + chest_eda + chest_emg	RF	0.810 ± 0.046	0.646 ± 0.118	0.557	+0.090
PAMAP2	imu_hand + imu_ankle	LDA	0.769 ± 0.034	0.650 ± 0.053	0.565	+0.085
PAMAP2	imu_hand + imu_ankle	SVM (RBF)	0.794 ± 0.019	0.688 ± 0.043	0.634	+0.054
PAMAP2	imu_hand + imu_ankle	RF	0.824 ± 0.009	0.732 ± 0.031	0.649	+0.083
PAMAP2	imu_hand + imu_ankle + heart_rate	LDA	0.788 ± 0.026	0.674 ± 0.050	0.565	+0.109
PAMAP2	imu_hand + imu_ankle + heart_rate	SVM (RBF)	0.813 ± 0.014	0.713 ± 0.040	0.634	+0.079
PAMAP2	imu_hand + imu_ankle + heart_rate	RF	0.830 ± 0.019	0.740 ± 0.026	0.649	+0.090

**Table 9:** Stage-wise covariance regularisation, hardware adaptation and deployment-oriented robustness summary.

Comparison Block	Model/Setting	Accuracy	Macro-F1	Interpretation
Stage I public covariance estimation (13-feature shared blocks)	LDA	0.243	0.243	Public covariance-estimation stage across modality-matched datasets; label spaces remain dataset-specific
Stage I covariance estimation (13-feature shared blocks)	RF	0.507	0.507	Public-only per-dataset RF baseline; incompatible class spaces across repositories—no cross-dataset label merging
Stage II hardware adaptation, 13-feature intersection	Transfer LDA	0.957	0.954	Positive λ-selected covariance regularisation using modality-matched public priors
Stage II hardware adaptation, 13-feature intersection	Scratch LDA	0.955	0.952	Hardware-only fit under shared intersection space
Stage II hardware adaptation, 13-feature intersection	Transfer RF	0.883	0.881	Null transfer outcome; independently fitted hardware RF under class-space mismatch
Stage II hardware adaptation, 13-feature intersection	Scratch RF	0.883	0.881	Hardware-only RF fit
Stage IIb full deployment schema, 17-feature RCG	Transfer LDA	0.952	0.948	Full-schema covariance-regularised hardware model
Stage IIb full deployment schema, 17-feature RCG	Scratch LDA	0.947	0.944	Hardware-only LDA under full 17-feature schema
Stage IIb full deployment schema, 17-feature RCG	Transfer RF	0.879	0.878	Schema-consistent hardware fit; no transferred class structure
Stage III deployment comparison	Edge LDA	0.947	0.944	716-byte embedded artefact with direct firmware export
Stage III deployment comparison	Cloud RF	0.880	0.879	Highest cloud-resident performance under the full 17-feature schema

**Table 10:** Final deployment-oriented comparison across the principal edge and cloud artefacts.

Deployment Target	Model	Active Schema	Accuracy	Macro-F1	Resource Note	Interpretation
Edge	LDA	17-feature full RCG schema	0.9470 ± 0.0258	0.9435 ± 0.0246	~716 bytes RAM	Primary embedded inference artefact
Edge	Quantised RF	17-feature full RCG schema	0.8329 ± 0.1593	0.8184 ± 0.1637	~7440 bytes RAM	Lower accuracy and F1, 10.4× larger footprint
Cloud	RF (200 trees, depth 15)	17-feature full RCG schema	0.8799 ± 0.1348	0.8792 ± 0.1313	Unconstrained cloud deployment	Retained cloud-side analytics artefact

## Data Availability

The data that support the findings of this study are not publicly available as the data are part of an ongoing study; the evaluation dataset comprises proprietary firmware configurations, cloud infrastructure parameters, and controlled bench evaluation session logs; and access is restricted to protect confidential and proprietary information.
